# Evolutionary dynamics and virulence factor variability in invasive *Streptococcus pyogenes* in Norway, 2017−2023

**DOI:** 10.1128/msphere.00775-25

**Published:** 2026-03-11

**Authors:** Ola Brønstad Brynildsrud, Hilde S. Vollan Gjerdrum, Einar Sverre Berg, Astrid L. Wester, Dominique A. Caugant

**Affiliations:** 1 Department of Method Development and Analytics, Division of Infection Control, Norwegian Institute of Public Health 25563, Oslo, Norway; 2 Department of Bacteriology, Division of Infection Control, Norwegian Institute of Public Health 25563, Oslo, Norway; 3 Department of Microbiology and Infection Control, Akershus University Hospital 60483, Lørenskog, Norway; 4 Department of Community Medicine, Faculty of Medicine, University of Oslo 6305, Oslo, Norway; McMaster University, Hamilton, Ontario, Canada

**Keywords:** iGAS, *emm *types, epidemiology, virulence genes, virulence factors

## Abstract

Invasive infections caused by *Streptococcus pyogenes* (iGAS) have increased in Europe over the past decade, with a marked upsurge after the COVID-19 pandemic. Here, we examined whether the increase in iGAS infections in Norway was associated with the spread of variants that had acquired new virulence factors. A collection of 1,163 iGAS isolates submitted to the National Reference Laboratory between January 2017 and April 2023, representing 87% of all cases recorded by the Norwegian Surveillance System for Infectious Diseases, was analyzed by whole genome sequencing. Resistance to one or more antibiotics was found in 15.6% of the isolates: 14.1% were resistant to tetracycline, 6.4% to erythromycin, and 4.0% to clindamycin. Resistance to other antibiotics was < 1%. The dominating *emm* types were *emm1* (30.9%), *emm12* (13.8%), *emm89* (9.3%), *emm28* (8.3%), *emm4* (6.0%), and *emm87* (4.5%), with the remaining isolates belonging to 55 other *emm* types; 62.3% of *emm1* belonged to the hypervirulent lineage M1_UK_. Genetic characterization of the virulence factors of the dominant six *emm* types demonstrated extensive competition between related phages, leading to phage switching and interplay between integration and excision of temperate phages carrying virulence factors. Bacteriophages carrying virulence factors *speC* and *spd1* displayed a particularly high turnover rate, with several pairs of otherwise genomically identical isolates exhibiting different phage-carrying status. We identified and characterized four new *speC* and *spd1*-carrying phages. The rapid turnover pattern of these, as well as other phages carrying superantigens and DNAses suggests an important role in pathogenesis.

**IMPORTANCE:**

This analysis of 1,163 iGAS isolates collected between January 2017 and April 2023 aimed to map virulence factor content to understand the observed increased incidence of iGAS in Norway. Our findings indicate that 15.6% of the isolates were resistant to at least one antibiotic, with tetracycline resistance being the most common. The dominant *emm* types were *emm*1, *emm*4, *emm*12, *emm*28, *emm*87, and *emm*89, which together accounted for 72.7% of the isolates. The study highlights the dynamic nature of virulence factor-carrying temperate phages, particularly the ones carrying *speC* and *spd1*, which frequently integrate and excise within *emm* types. Four previously unseen phages carrying *speC* and *spd1* were identified and characterized. This research underscores the complexity of iGAS epidemiology and the need for continuous surveillance to understand the evolving landscape of bacterial virulence factors, antibiotic resistance, and circulating *emm* types.

## INTRODUCTION

*Streptococcus pyogenes*, also called group A *Streptococcus* (GAS), is a strictly human pathogen causing a wide spectrum of diseases, ranging from mild pharyngitis infections to severe invasive life-threatening diseases (invasive GAS, iGAS), such as necrotizing soft tissue infection (NSTI) and streptococcal toxic shock syndrome (STSS) (1). It has been estimated that there are over 600,000 iGAS infections globally and 160,000 deaths per year (2).

The M protein, a fibrillar protein that extends from the bacterial cell wall, serves as the basis for the classification of GAS. The first 180 base pairs of the gene encoding the M protein, the *emm* gene, are used for *emm* genotyping. There are over 275 distinct *emm* types identified globally, indicating that GAS exhibits remarkable genetic diversity and human adaptability (https://www.cdc.gov/strep-lab/php/group-a-strep/emm-typing.html).

It has been reported that the *emm* type distribution shows spatio-temporal patterns, with lower-income countries having higher GAS strain diversity (3, 4). The *emm1* gene, coding for the M1 protein, is one of the most frequent types isolated from invasive infections worldwide (5). Recently, a variant of M1 called M1_UK_ has emerged. The M1_UK_ lineage differs from the progenitor M1 strain by 27 single-nucleotide polymorphisms across the core genome and is associated with upsurges of both scarlet fever, invasive infections, and death in children (6). This lineage, which is estimated to have first appeared in 2008, rapidly became dominant in some regions, including Europe, North America, and Australia, and has shown evidence of enhanced fitness and virulence (6–11). The increased virulence of M1_UK_ is known to be at least partly driven by increased expression of the superantigen SpeA due to a single-base mutation in an upstream gene (7), but in general, there are gaps in our understanding of genetic factors that promote virulence heterogeneity between different GAS lineages.

In addition to the M protein, GAS exhibits a wide range of virulence factors, including proteases, toxins, and superantigens, which play crucial roles in pathogenesis by modulating the host immune response (2). For instance, the streptococcal superantigen (SSA), the streptococcal pyrogenic exotoxin A (SpeA), the streptococcal pyrogenic exotoxin C (SpeC), various prophage- and chromosome-encoded DNases, streptolysin O (SLO), streptolysin S (SLS), and NAD glycohydrolase (NADase) are all able to induce massive T-cell activation and cytokine release, contributing to the severity of GAS infections (2, 12, 13). The production of multiple superantigens provides GAS with mechanisms to promote its survival and transmission within human populations. Despite extensive efforts, this diversity and versatility of GAS strains represent a significant challenge for the development of an effective vaccine (14), although vaccine candidates have been proposed (https://www.who.int/teams/immunization-vaccines-and-biologicals/diseases/streptococcus-pyrogenes [sic]). As of 2023, there were eight vaccine candidates in development, the most advanced of which had completed phase 1 clinical trials (15).

The *hasABC* operon that encodes proteins that synthesize the hyaluronic acid capsule also plays an important role in GAS virulence (16), but not all GAS carry the operon, and some lineages do not express a capsule even if they do carry it. The GAS capsule mimics the human hyaluronic acid and enables the bacterium to evade the host immune responses (17). This operon has a regulatory sequence in the promoter that plays a crucial role in the regulation of capsule production through an interaction with the two-component regulatory system CovRS. CovRS regulates the production of multiple virulence factors (17), and *covS* mutations have been associated with the onset of severe invasive infections (18).

Whole genome sequencing (WGS) enables high-resolution insights into GAS strain diversity, virulence factor profiles, and antimicrobial resistance patterns (19). WGS analyses have become crucial for better understanding the epidemiology of GAS, for surveillance purposes, and informing public health interventions (20). In this study, we characterized iGAS isolates collected in Norway from January 2017 to April 2023, with a special focus on the six most prevalent *emm* types and their phylogenetic relationships. We explored how genes encoding known virulence factors varied within and between each of these six lineages. We documented the extensive plasticity in virulence factors that were associated with temperate phages, which suggests an ongoing evolutionary arms race between phages and bacterial hosts, as well as between different related phages that compete for the same binding site in the host. This study provides a comprehensive genomic epidemiological analysis of 1,163 iGAS in Norway and explores generalizable patterns of virulence factor evolution within the six most common *emm* types.

## RESULTS

### iGAS epidemiology in Norway

**Fig 1:**
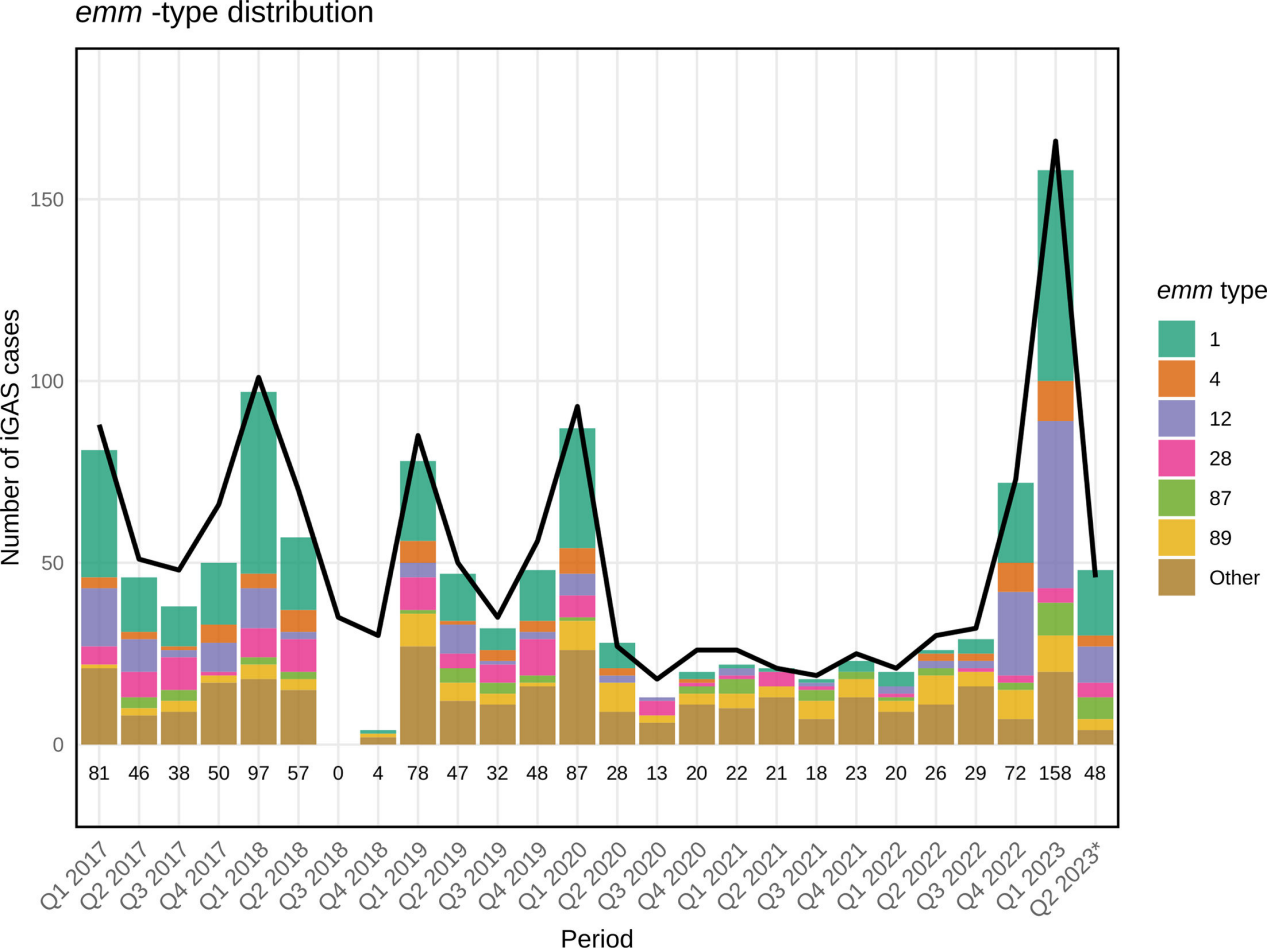
Number of sequenced iGAS per quarter between 2017 and 2023 by *emm* type. The black line represents the total number of iGAS cases reported to the Norwegian Surveillance System for Infectious Diseases (MSIS). *The study period ended 30 April 2023. The total number of samples for each quarter is given above the timeline.

Overall, 87% of the iGAS cases reported to the Norwegian Surveillance System for Infectious Diseases (MSIS) during the study period were successfully sequenced. Except for an interruption of sequencing in quarters Q3 and Q4 2018, the number of sequenced isolates correlated well with the number of reported cases (Fig. 1). The number of cases had a seasonal variation with a peak in Q1, except during the pandemic period (between Q2 2020 and Q1 2022). The age distribution of the patients with sequenced samples (Fig. S1) closely mirrored that of registered patients in the MSIS database. The average incidence rate was 3.8 per 100,000 person-years in the 0−9 years group, down to 0.8 in the 10−19 age group, and then steadily rising with age, up to 9.3 in the 70+ group. Metadata and accession numbers for the sequences of all 1,163 isolates can be found in Table S1.

### *emm* type distribution

Among the 61 *emm* types identified, *emm*1 was the most prevalent (30.9%), although its frequency declined during the pandemic; notably, 62.3% of *emm*1 isolates belonged to the hypervirulent M1_UK_ lineage. Other common types included *emm*12 (13.8%), *emm*89 (9.3%), *emm*28 (8.3%), *emm*4 (6.0%), and *emm*87 (4.5%), while the remaining 55 *emm* types were grouped as “Others” (27.3%); 20% of *emm*4 belonged to the recently described hypervirulent M4_NL22_ clade (21). The relative distribution of *emm* types according to region, age group, gender, season, and the period of the pandemic is detailed in Table 1. The relative frequency of the hypervirulent lineage M1_UK_ compared to M1_global_ (non-M1_UK_ *emm1*) did change significantly during the study period, with a binomial regression model indicating an increase from 37% (95% CI: 29%−50%) in 2017 to 63% (95% CI: 57%−67%) in 2023.

**TABLE 1:** Distribution of common *emm* types of invasive *S. pyogenes* in Norway, 2017−2023 by region, age group, gender, and seasonality^*a*^

	*emm1* (*N* = 359)	*emm4* (*N* = 70)	*emm12* (*N* = 160)	*emm28* *N* = 96)	*emm87* (*N* = 52)	*emm89* (*N* = 108)	Others (*N* = 318)	Total iGAS (*N* = 1,163)	Norway (*N* = 5.3 million)
Region									
East	55.4%	52.9%	50.6%	***41.7%***	50.0%	59.3%	**68.2%**	57.1%	50.7%
South	3.9%	5.7%	6.9%	7.3%	1.9%	3.7%	3.7%	4.8%	5.7%
West	18.7%	21.4%	26.9%	27.1%	**38.5%**	17.6%	***14.5%***	20.3%	25.8%
Mid	12.3%	14.3%	9.4%	13.5%	0.0%	13.0%	15.9%	10.2%	8.7%
North	9.7%	5.7%	6.2%	10.4%	9.6%	6.5%	5.7%	7.7%	9.1%
Age									
0−9 years	**17.0%**	15.7%	**25.6%**	10.4%	9.6%	10.2%	***2.2%***	12.6%	11.4%
10−19 years	3.9%	0.0%	1.2%	0.0%	1.9%	4.6%	3.1%	2.8%	12.0%
20−39 years	***7.5%***	20.0%	***8.8%***	24.0%	21.2%	20.4%	**29.2%**	17.5%	26.8%
40−69 years	**40.4%**	27.1%	32.5%	22.9%	36.5%	***22.2%***	36.5%	34.1%	37.5%
>70 years	31.2%	37.1%	31.9%	42.7%	30.8%	42.6%	28.9%	33.0%	12.3%
Sex									
Female	46.8%	54.3%	**37.5%**	59.4%	50.0%	53.7%	43.7%	46.9%	50.0%
Other									
Pandemic	***11.5%***	1.8%	***4.8%***	7.3%	7.3%	**20.0%**	**47.3%**	7.0%	NA
Winter	**34.5%**	6.3%	**15.8%**	***6.3%***	3.4%	7.8%	25.9%	65.6%	NA

^*a*^ Categories with significantly higher (underlined) or lower (italicized) occurrences of a particular *emm* type, as compared to all *emm* types in that category, are in bold (based on FDR-adjusted *P*-values from logistic regression analysis). The last column shows the percentage of the Norwegian population constituting each category (https://www.ssb.no/statbank/table/07459). NA denotes that data are not applicable for the variables Pandemic and Winter, as these factors are not part of the demographic data obtained from Statistics Norway (SSB).

### Antimicrobial resistance

Among the 854 isolates with antimicrobial susceptibility testing (AST) data, 135 (15.8%) displayed resistance to one or more antibiotics, with 34 isolates (4.0%) displaying some level of resistance toward clindamycin (minimum inhibitory concentration [MIC] >0.5), 57 (6.7%) toward erythromycin (MIC >0.25), 123 (14.4%) toward tetracycline (MIC >1), and four isolates (0.5%) toward trimethoprim-sulfamethoxazole (MIC >1). In total, 51 isolates (6.1%) were resistant to two or more antibiotics, most commonly tetracycline and macrolides, lincosamides, and streptogramin (MLS) (tetracycline/erythromycin, 48 [5.6%]; tetracycline/clindamycin, 33 [3.9%]; tetracycline/clindamycin/erythromycin, 32 [3.7%]; clindamycin/erythromycin without tetracycline, 2 [0.2%]). No isolates displayed phenotypic resistance towards penicillin G.

### Correlation between phenotypic and genotypic resistance

The correlation between phenotypic resistance and individual antimicrobial resistance (AMR)-associated genes is shown in Table 2. Antimicrobial resistance in GAS is often associated with integrative conjugative elements (ICEs) (22), but due to high levels of mosaicism and repeated sequence, these are often untypable with short-read data. Having both phenotypic AST data and genetic predictions, we set out to determine how well genetic data predicted phenotypic resistance in *S. pyogenes*. For erythromycin resistance, we counted *ermA*, *ermB*, *ermT*, *mefA,* and *msrD* as contributing. Both the sensitivity and the positive predictive value (PPV) of the prediction was 100.0% (57/57). The erythromycin resistance-associated genes were *ermA* (*N* = 12), *ermB* (*N* = 36), *ermT* (*N* = 1), or *mefA* and *msrD* (always appearing together, *N* = 8). No isolates had erythromycin resistance from multiple sources, for example, both *ermA* and *ermB*, or any *erm* gene together with *mefA*/*msrD*.

**TABLE 2:** Antimicrobial resistance genes identified in phenotypically resistant isolates^*a*^

AMR gene	Clindamycin resistance	Erythromycin resistance	Tetracycline resistance	Trimethoprim sulfa resistance	*emm* types
ant(6)-Ia	1 (>256)	2 (>256)	1 (32)	1 (32)	28.0, 49.0
aph(3')-III	6 (>256)	8 (4−>256)	7 (8−64)	1 (32)	11.0, 11.17, 28.0, 49.0, 77.4, 81.0
dfrG	0 (-)	0 (-)	1 (16)	1 (8)	104.0
erm(A)	1 (>256)	12 (1−8)	11 (4−64)	0 (-)	58.0, 73.0, 77.0, 77.4, 89.0, 94.1
erm(B)	32 (>256)	36 (1−>256)	34 (2−64)	1 (32)	102.2, 102.3, 11.0, 11.1, 11.17, 12.0, 169.3, 27.0, 28.0, 48.1, 49.0, 66.1, 73.0, 76.0, 81.0, 82.0, 89.0
erm(T)	0 (-)	1 (>256)	0 (-)	0 (-)	4.0
lsa(C)	1 (1)	0 (-)	1 (32)	0 (-)	118.2
mef(A)	0 (-)	8 (4−64)	3 (32−>256)	0 (-)	12.0, 183.2, 2.0, 4.0, 44.2, 63.0, 9.0
msr(D)	0 (-)	8 (4−64)	3 (32−>256)	0 (-)	12.0, 183.2, 2.0, 4.0, 44.2, 63.0, 9.0
tet(L)	0 (-)	1 (8)	3 (64−>256)	0 (-)	169.0, 44.2, 68.12
tet(M)	32 (1−>256)	41 (1−>256)	113 (2−>256)	2 (8−32)	102.2, 102.3, 104.0, 106.0, 11.0, 11.1, 11.17, 110.0, 118.2, 12.0, 169.0, 169.3, 169.5, 183.1, 183.2, 203.4, 22.0, 249.0~, 25.1, 27.0, 28.0, 4.5, 41.8, 41.9, 44.2, 48.1, 49.0, 49.2, 58.0, 63.0, 66.0, 66.1, 68.12, 68.3, 73.0, 75.0, 76.0, 77.4, 79.5, 81.0, 82.0, 83.1, 88.2, 89.0, 90.2, 94.1
tet(O)	0 (-)	6 (1−2)	6 (4−64)	0 (-)	77.0
tet(S/M)	0 (-)	0 (-)	1 (32)	0 (-)	59.0
tet(T)	0 (-)	1 (4)	2 (4−64)	0 (-)	226.0, 73.0

^*a*^ Number of phenotypically resistant isolates harboring antimicrobial resistance-associated genes, including corresponding *emm* types. Minimum inhibitory concentration ranges are shown in parentheses (lowest–highest).

For lincosamide and clindamycin resistance, we counted *ermA* (*N* = 12), *ermB* (*N* = 36), *ermT* (*N* = 1), and *lsaC* (*N* = 1) as resistance genes. The sensitivity was 100% (34/34), but the PPV was lower, as only 68.0% (34/50) of isolates carrying these genes were phenotypically resistant towards clindamycin (MIC > 0.5). While all isolates with the *lsaC* gene displayed phenotypic resistance toward clindamycin, the individual-gene PPV for predicting clindamycin resistance was 88.9% (32/36) for *ermB*, but only 8.3% (1/12) for *ermA* and 0.0% (0/1) for *ermT*. The low PPV for *ermA* and *ermT* is expected, since these genes only provide inducible resistance to the MLS family of antibiotics, meaning that gene expression is usually low without prior exposure to erythromycin. When using only *ermB* and *lsaC* as predictors for clindamycin resistance, the sensitivity was 100% (34/34) and the PPV 89.2% (33/37).

For tetracycline, the sensitivity of the prediction was 99.2% (122/123), and the PPV was 98.4% (122/124). The most common tetracycline resistance genes were *tetM* (*N* = 113), *tetO* (*N* = 6), *tetL* (*N* = 3), and *tetT* (*N* = 2).

For trimethoprim-sulfamethoxazole, predictions were much poorer. Out of four isolates that were resistant to AST, only one isolate carried the *dfrG* gene, indicating a predicted sensitivity of 25.0% (1/4). The PPV was 100% (1/1), however.

### Variation in resistance over time and between *emm* types

The fraction of resistant isolates was relatively stable during the study period but had a peak in 2021, with 38/85 isolates (38.8%) being resistant to one or more antibiotics. Although iGAS incidence was lower during the pandemic, this period saw a relative increase in frequency of highly resistant *emm* types like *emm11*, *emm22,* and *emm106* (23).

Resistance varied extensively by *emm* type, as shown in the phylogenetic tree over all 1163 isolates in Fig. S2. It was low in the major six *emm* types. In *emm1*, a single isolate was resistant to macrolides (1/359; 0.3%) and none to other antibiotics. In *emm4*, five isolates were resistant to macrolides and one to tetracycline, meaning 8.3% were resistant to one or more antibiotics (6/70). In *emm12*, one was resistant to macrolides and one to MLS and tetracycline, for a total of 1.3% resistant isolates (2/160). In *emm28*, one isolate was resistant to MLS and aminoglycosides (not confirmed by AST), one to macrolides and tetracycline, and one to tetracycline and trimethoprim-sulfamethoxazole, for a total of 3.1% (3/96) resistance. There were no resistant isolates within *emm87*. For *emm89*, one isolate was mono-resistant to tetracycline, two to MLS and tetracycline, one to macrolides only, and one to trimethoprim-sulfamethoxazole, for a total of 4.6% (5/108) resistant isolates. The highest fractions of resistant isolates (among *emm* types with more than 20 isolates) were found in *emm11* (27/27; 100%), *emm106* (22/22; 100%), and *emm118* (31/32; 96.9%).

### Genomic distribution of virulence factors

#### Core virulence factors

A list of 66 virulence factors that were tested for and their distribution patterns across the different *emm* types can be found in Table 3. Some virulence factors were ubiquitous, found in all isolates in this study. They appear to represent core genes under strong purifying selection pressure and are likely necessary for producing infection. They can further be divided into genes that were always found as full-length ORFs and genes that would sometimes be present as pseudo-genes. This indicates that *S. pyogenes* can, in some cases, cause invasive infection without these virulence factors or, alternatively, that these variants arise and proliferate over the course of invasive infection but are rarely fit enough to propagate to new patients.

**TABLE 3:** Presence and absence of virulence factor-encoding genes across the six major *emm* types^*a*^

Name	Alt. name(s)	Ref. accession	Description	C/P	Core	Presence in *emm* type						Ref
						1	4	12	28	87	89	
Superantigen exotoxins												
*speA*		WP_009880239	Superantigen	P		99.7	-	4.3	-	-	-	(24)
*speC*		WP_021733756	Superantigen	P		7.2	+	96.3	+	71.2	85.2	(24)
*speG*		WP_038431202	Superantigen	C		+*	-	+	+	+	+	(24)
*speH*		WP_010922229	Superantigen	P		-	-	98.8	1.0	1.9	1.9	(24)
*speI*	*entE*	WP_047373492	Superantigen	P		-	-	98.8	1.0	1.9	1.9	(24)
*speJ*		WP_010921949	Superantigen	C		+	-	-	+	98.2*	-	(24)
*speK*		WP_002983113	Superantigen	P		+	+	+	+	+	+*	(24)
*speL*		WP_020833516	Superantigen	P		-	-	-	-	-	1.9	(24)
*speM*		WP_038431384	Superantigen	P		-	-	-	-	-	-	(24)
*speQ*		WP_085634345	Superantigen	C		-	-	-	-	+	-	(25)
*speR*		WP_269458603	Superantigen	C		-	7.1*	-	-	+	-	(25)
*smeZ*		WP_023610175	Superantigen	C		+	+	+	+	+	+	(24)
*ssa*	*entC3*	WP_002988472	Enterotoxin type C3	P		0.3	+	26.8	-	+	-	(26)
Other exotoxins												
*speB*		WP_014407896	Cysteine protease B	C	Y	+	+	+	+	+	+	(27)
*slo*	Streptolysin O	WP_080281942	Pore formation in host cells	C	Y	+	+	+	+	+	+	(28)
*sagA*	Streptolysin S	WP_002985285	Pore formation in host cells	C	Y	+	+	+	+	+	+	(29)
*nga*		WP_021340669	Exotoxin A, NAD+ glycohydrolase	C	Y	+	+	+	+	+	+	(30)
*Ifs*		WP_002987884	NADase inhibitor, *nga* antitoxin	C	Y	+	+	+	+	+	+	(31)
*cfa*		WP_009880829	Pore-forming toxin	C		+	+	+	+*	+	+	(32)
Capsule												
*hasA*		WP_038431685	Hyaluronan synthase	C		+*	-	+*	*	*	1.9	(33)
*hasB*		WP_111679870	Part of capsule formation	C		+*	-	+*	95.8	+*	1.9	(33)
*hasC*		WP_002982024	Non-essential part of capsule	C		+	-	+	+	+	1.9	(33)
*hyl*		WP_011054788	Hyaluronate lyase precursor	C	Y	+	+	+	+	+	+	(34)
HasS		-	Small RNA affecting *hasABC*	C		Δ9	wt	wt†	-	-	-	(17)
Adhesins												
*cpa*		WP_168639774	Collagen-binding protein A	C		+	-	-	-	-	-	(35)
Spy0128		WP_346393375	Pilin	C		-	-	+	+	+	+	(36)
*sfb1*	*prtf1*, protein F1	WP_041174256	Fibronectin-binding protein 1	C		-	+	+	+	+	+	(37)
*sfb2*	*prtf2*, protein F2	WP_136299195	Fibronectin-binding protein 2	C		-	-	+	+	+	+	(37)
*fbpA*	*fbaA*	WP_014635747	Fibronectin-binding protein A	C		+	+	-	+	+	+	(37)
*scl1*	*sclA*	WP_111679864	Collagen-like surface protein	C	Y	+	+	+	+	+	+	(38)
*scl2*	*sclB*	WP_369324857	Collagen-like surface protein	C	Y	+	+	+	+	+	+	(38)
Lmb	*lbp, znuA*	WP_002987954	Zinc receptor, laminin binding	C	Y	+	+	+	+	+	+	(38)
R28	*bca*	WP_011285020	Epithelial adhesion	C		-	-	-	+	-	-	(39)
*eno*	*sen*	WP_023078153	Plasminogen binding protein	C	Y	+	+	+	+	+	+	(40)
Immune modulators												
*sic*		AAF34323.2	Complement inhibitor	C		+	-	-	-	-	-	(41)
*ska*	Streptokinase	WP_038431609	Plasminogen-activating protein	C	Y	+	+	+	+	+	+	(42)
*emm*	M protein	WP_111679868	Binds a range of host proteins	C	Y	+	+	+	+	+	+	(43)
*mrp*		WP_014407887	M-related protein, binds IgG	C		-	+	-	+	+	+	(44)
*enn*		WP_111679867	M-like protein, binds IgA	C		-	+	-	+	+	+	(45)
*ess*	S protein	WP_002990270	Antiphagocytic mimicry of RBC	C	Y	+	+	+	+	+	+	(46)
*spyCEP*	*spcP, cep*	WP_174145361	Inactivate neutro. chemoattractants	C	Y	+	+	+	+	+	+	(47)
*scpA*		WP_011285740	C5a peptidase	C	Y	+	+	+	+	+	+	(48)
*ideS*	Mac-1, Mac-2	WP_010922160	IgG specific degradation	C	Y*	+	+*	+*	+	+	+	(49)
*ndoS*		WP_011285695	IgG specific degradation	C	Y*	+	+	+*	+*	+	+	(50)
Deoxyribonucleases												
*sda1*	*sdaD*	WP_011018105	Streptodornase	P		-	-	-	-	-	-	(51)
*sda2*	*spd2, sdaD2*	WP_136304021	Streptodornase D	P		96.4	-	96.9	3.1	-	1.9	(51)
*sda3*	*spd3, mf3*	WP_011285611	Streptodornase	P		+	+	2.5	-	+	47.2	(51)
*spd1*	*mf2, sdaC*	WP_002985324	Streptodornase C	P		7.2	+	96.3	+	71.2	85.2	(51)
*spd4*		WP_002983479	Non-specific endonuclease	P		0.3	-	-	-	-	-	(51)
*sdn*	*sda, sdaA*	WP_050440680	Streptodornase A	P		-	45.7	1.3	4.2	96.2	8.3	(51)
*spnA*		WP_002985259	Endonuclease	C	Y	+	+	+	+	+	+	(51)
*sdaB*	*speF, mf1, spdB*	WP_010922721	Streptodornase B	C	Y	+	+	+	+	+	+	(51)
Transcriptional regulators												
*covR*	*csrR*	WP_002991052	Virulence regulator (with *covS*)	C	Y	+	+	+	+	+	+	(52)
*covS*	*csrS*	WP_002991036	Virulence regulator (with *covR*)	C	Y*	+*	+*	+*	+*	+	+*	(52)
*rocA*		WP_011285039	Global virulence regulator	C	Y*	+	+*	+	+	+*	+*	(53)
*fabT*	*marR, pchR*	WP_002993768	Fatty acid biosynthesis regulator	C	Y	+	+	+	+	+	+	(54)
*mtsR*	*mntR*	WP_011285412	Metal transport system regulator	C	Y	+	+	+	+	+	+	(55)
*trxR*	*rhaR*	WP_010922520	CovR-repressed regulator of Mga	C	Y	+	+	+	+	+	+	(56)
*nga-ifs-slo*		-	Promoter of *nga-ifs-slo* locus	C	Y	3-1	3-1	3-1	1-1	3-2	3-1	(57)
RNA modification												
*rnc*		WP_002990670	Ribonuclease III	C	Y	+	+	+	+	+	+	(58)
*rny*		WP_002988954	Ribonuclease Y	C	Y	+	+	+	+	+	+	(59)
*rnr*		WP_080281949	Ribonuclease R	C	Y	+	+	+	+	+	+	(60)
*yhaM*		WP_002986055	Exoribonuclease	C	Y	+	+	+	+	+	+	(60)
*pnpA*		WP_000845143	Polynucleotide phosphatase	C		-	-	-	-	-	-	(60)
Other												
*hdsM*	*hsdM*	WP_010922657	Restriction-modification system	C	Y	+	+	+	+	+	+	(61)
Shr		WP_111679827	Heme receptor	C	Y*	+	+	+	+	+*	+	(62)

^*a*^ C/P, chromosome/prophage associated. Core: Y, in all genomes in this study (not just the six major *emm* types); Y*, in all genomes, but sometimes as pseudogene. Presence in *emm* type: +, present in all; -, absent in all; number, percentage of isolates with gene present; *, pseudogene variants exist. HasS: wt, wild type; Δ9, 9 bp deletion in terminator; wt†, multiple types of indels; -, absent/irrelevant. *nga-ifs-slo*, types 3 and 4 are associated with higher expression.

Virulence factors that were always present as full-length ORFs included streptolysins S (*sagA*), streptolysin O (*slo*), cysteine protease (*speB*), exotoxin A/NAD+ glycohydrolase *nga*, the *nga* antitoxin NADase inhibitor (*ifs*), hyaluronate lyase precursor (*hyl*), the streptococcal collagen-like surface proteins (*scl1*, *scl2*), laminin-binding adhesin (*lmb*/*znuA*), plasminogen binding protein (*eno*), streptokinase (*ska*), M protein (*emm*), S protein (*ess*), chemokine-inactivating protease (*spyCEP*), C5a peptidase (*scpAB*), streptodornase B (*sdaB*/*speF*), and NADase (*spnA*). Additionally, all isolates contained a set of genes not considered classical virulence factors, but associated with virulence. They included transcriptional regulators such as the responder part of the two-component regulator of virulence (*covR)*, the multigene regulator (*mga)*, fatty acid biosynthesis transcriptional regulator (*fabT)*, metal transport system regulator (*mtsR)*, and the CovR-repressed response regulator of Mga (*trxR)*, RNA modifiers ribonuclease III, Y, and R (*rnc*, *rny*, *rnr*), exoribonuclease (*yhaM)*, and a restriction modification system (*hdsM*). Virulence factor-encoding genes that were always present but that had occasionally formed into pseudogenes through indel mutations included the sensor part of the two-component regulator of virulence (*covS*), the global virulence regulator (*rocA)*, IgG-degrading enzymes (*ideS*/Mac-1/Mac-2 and *ndoS*), and the streptococcal heme receptor (*shr*). These pseudogene mutations were found in singular or closely related isolates, indicating that these genotypes are likely associated with some fitness loss and do not efficiently spread to new patients.

#### Accessory virulence factors and their distribution by *emm* type

**Fig 2:**
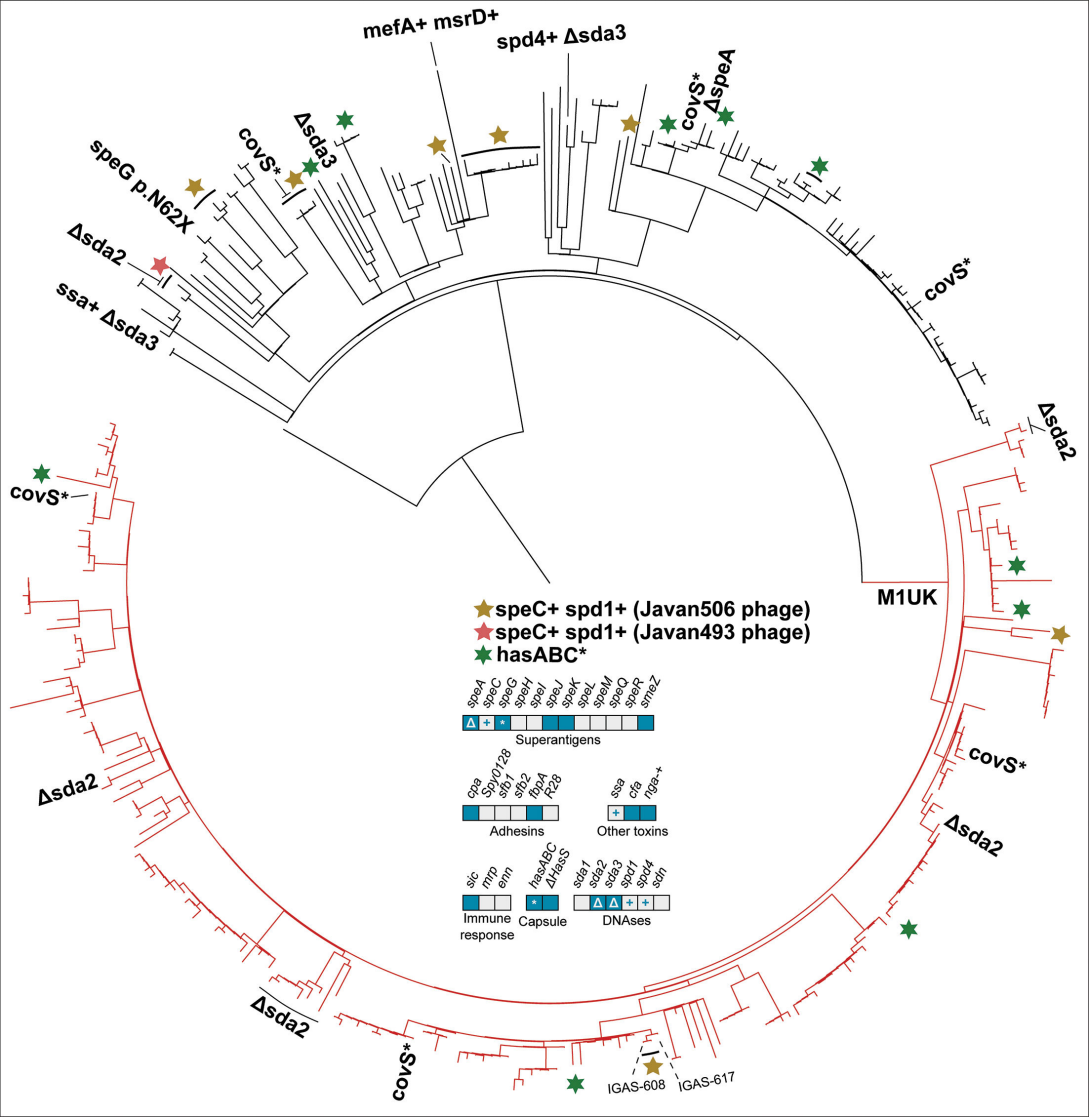
Phylogenetic tree of *emm*1 isolates from Norway, 2017−2023. Variations in accessory virulence factor content are annotated on branches, with lines perpendicular to the ring drawn to include multiple isolates where mutations are not just in singular isolates. A “+” indicates acquisition of a gene, and “Δ” a loss. The central panel indicates the the presence/absence of accessory virulence genes in *emm1*. Blue boxes indicate the presence and gray boxes indicate the absence of a virulence factor-encoding gene. A gray box with a “+” indicates sporadic gain, while a blue box with a “Δ” indicates sporadic loss. A “*” indicates sporadic pseudogenization. M1_UK_ is resolved as a monophyletic group on the red part of the tree. Most of *emm1* lacked a *speC-spd1*-carrying phage, but some isolates carried the Javan506 phage (yellow star) and some the Javan493 (red star). Pseudogene mutations in *hasABC* were common in *emm1*, annotated with a green six-pointed star. Specific isolates referenced in the manuscript are named on the tree.

**Fig 3:**
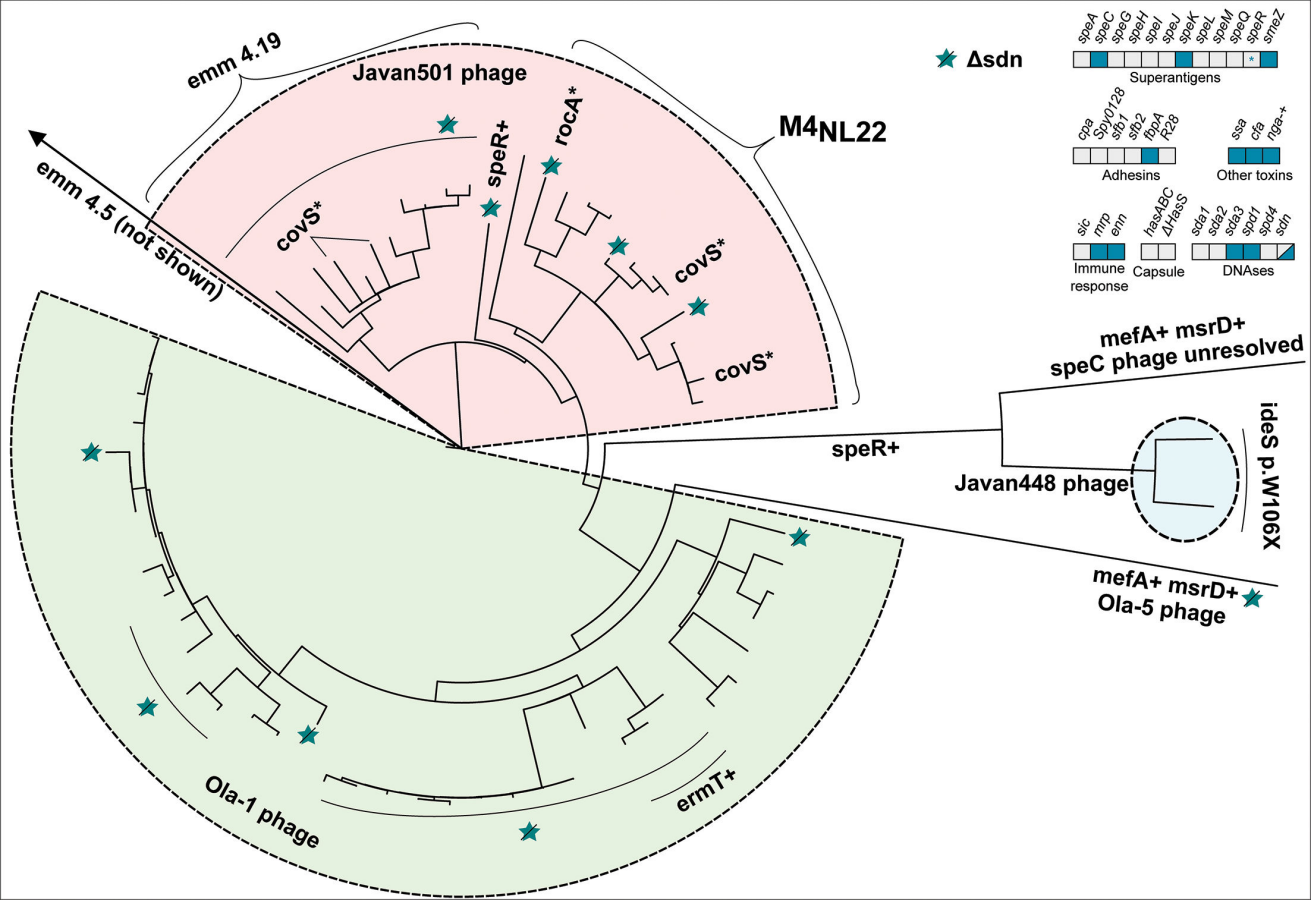
Phylogenetic tree of *emm*4 isolates from Norway, 2017−2023. Variations in accessory virulence factor content are annotated on branches, with lines perpendicular to the ring drawn to include multiple isolates where mutations are not just in singular isolates. A “+” indicates acquisition of a gene, and “Δ” a loss. The top right panel indicates the presence/absence of accessory virulence genes in *emm4*. Blue boxes indicate the presence and gray boxes indicate the absence of a virulence factor-encoding gene. Mixed blue and gray indicates variable presence. A gray box with a “+” indicates sporadic gain, while a blue box with a “Δ” indicates sporadic loss. A “*” indicates sporadic pseudogenization. M4_NL_ and *emm*4.19 are both resolved as monophyletic groups. Five different *speC-spd1*-carrying phages were seen in *emm4*, with the majority having either Ola-1 (green background) or Javan501 (red background). Two isolates carried Javan448 (blue background), whereas one carried Ola-5 and one an unresolved *speC-spd1*-phage. The loss of an unresolved *sdn*/streptodornase-carrying phage was common across the phylogeny, indicated by a teal star with a line across. A single *emm4.5* was only distantly related and not shown on the phylogeny.

**Fig 4:**
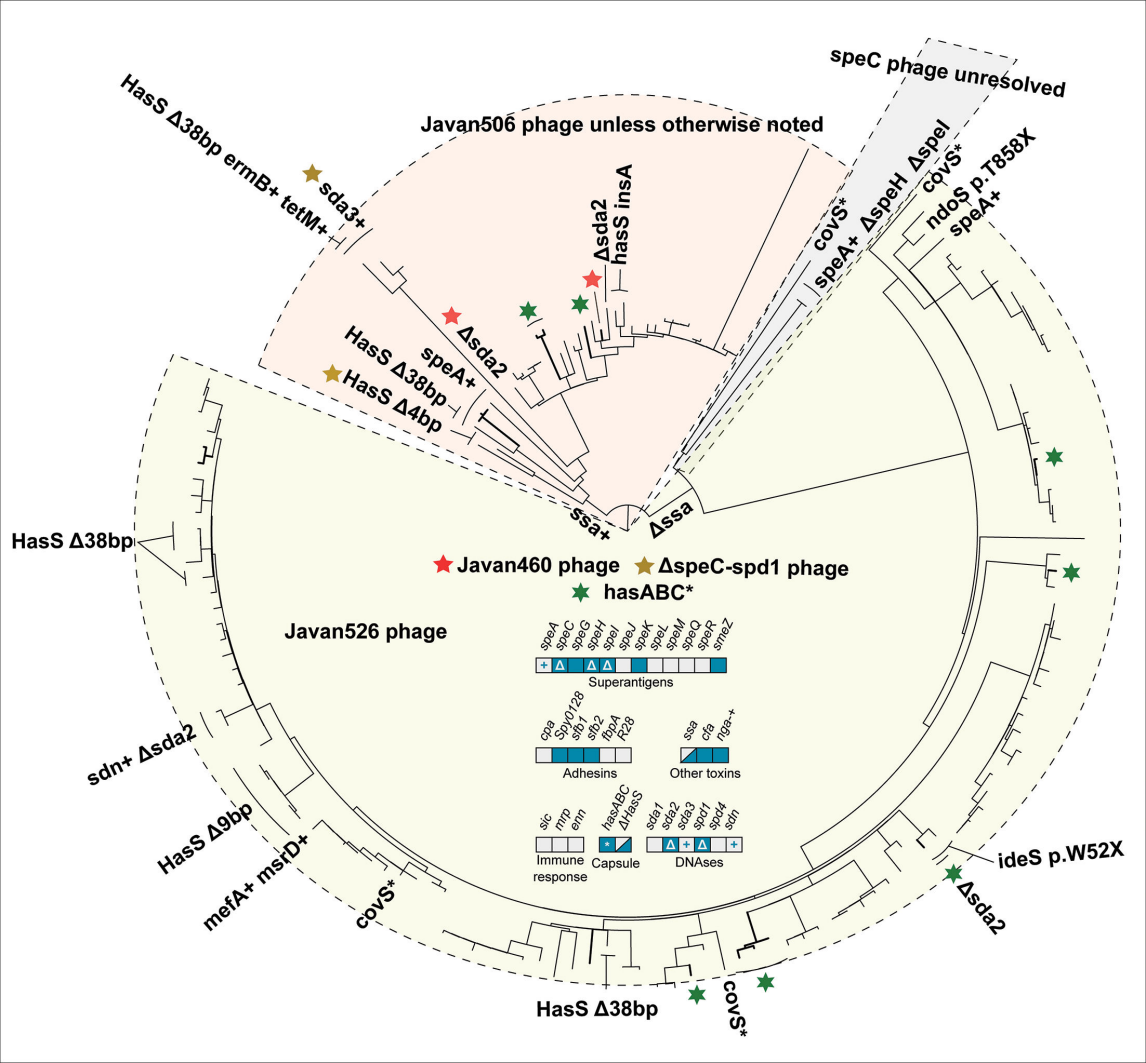
Phylogenetic tree of *emm*12 isolates from Norway, 2017−2023. Variations in accessory virulence factor content are annotated on branches, with lines perpendicular to the ring drawn to include multiple isolates where mutations are not just in singular isolates. A “+” indicates acquisition of a gene, and “Δ” a loss. The central panel indicates the presence/absence of accessory virulence genes in *emm12*. Blue boxes indicate the presence and gray boxes indicate the absence of a virulence factor-encoding gene. Mixed blue and gray indicates variable presence. A gray box with a “+” indicates sporadic gain, while a blue box with a “Δ” indicates sporadic loss. A “*” indicates sporadic pseudogenization. A majority of *emm12* isolates carried the Javan526 phage (tan background), and roughly one-fifth carried Javan506 (red background). A few isolates also carried Javan460 (red star), or no *speC-spd1* phage at all (yellow star). The phage was unresolvable in three isolates (gray background). Capsule locus pseudogenization is indicated with green six-pointed stars.

**Fig 5:**
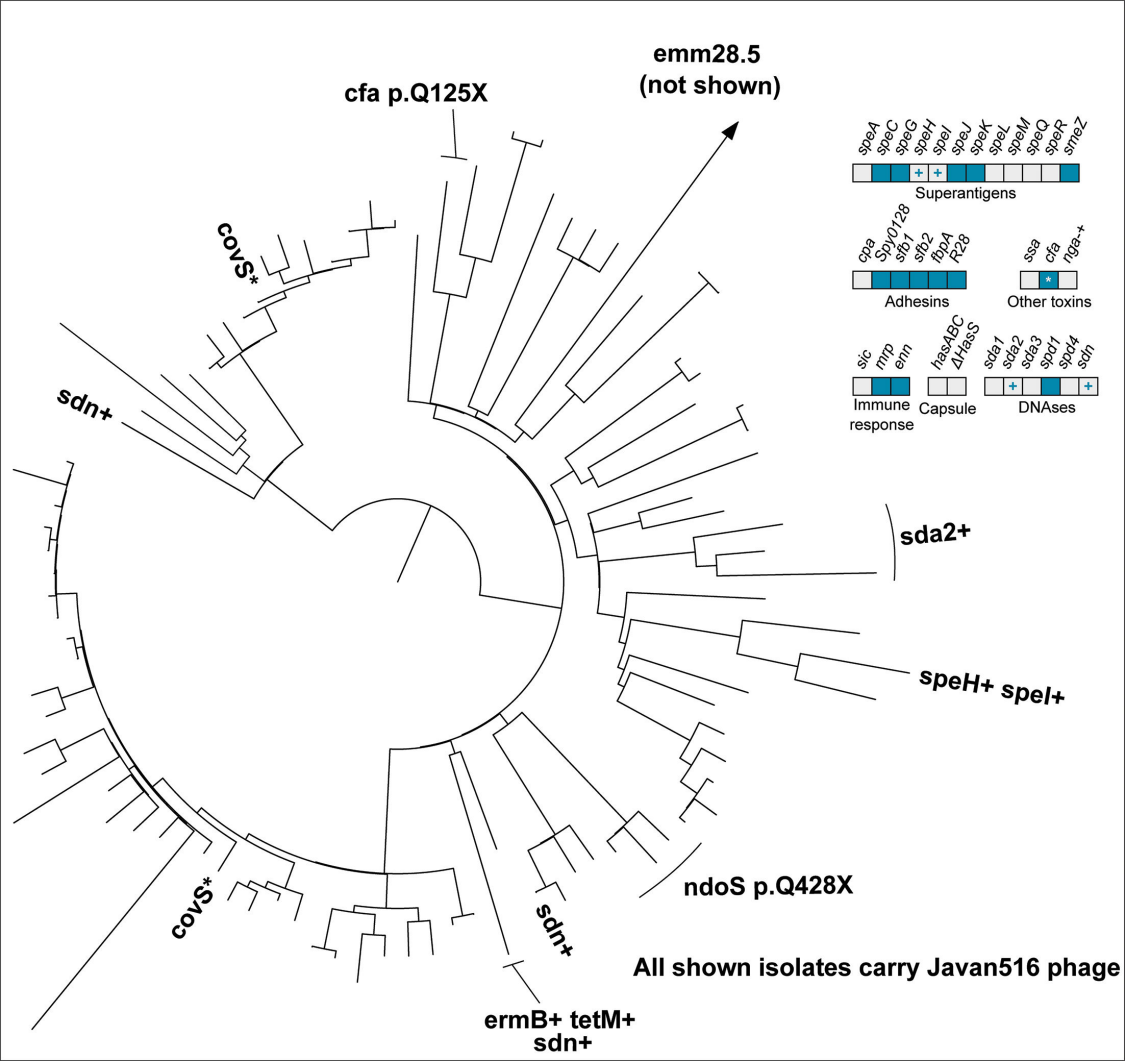
Phylogenetic tree of *emm*28 isolates from Norway, 2017−2023. Variations in accessory virulence factor content are annotated on branches, with lines perpendicular to the ring drawn to include multiple isolates where mutations are not just in singular isolates. A “+” indicates acquisition of a gene. The top right panel indicates the presence/absence of accessory virulence genes in *emm28*. Blue boxes indicate the presence and gray boxes indicate the absence of a virulence factor-encoding gene. Mixed blue and gray indicates variable presence. A gray box with a “+” indicates sporadic gain. A “*” indicates sporadic pseudogenization. A single *emm28.5* was distantly related and not shown on the tree. All isolates carried the *speC-spd1*-carrying Javan516 phage.

**Fig 6:**
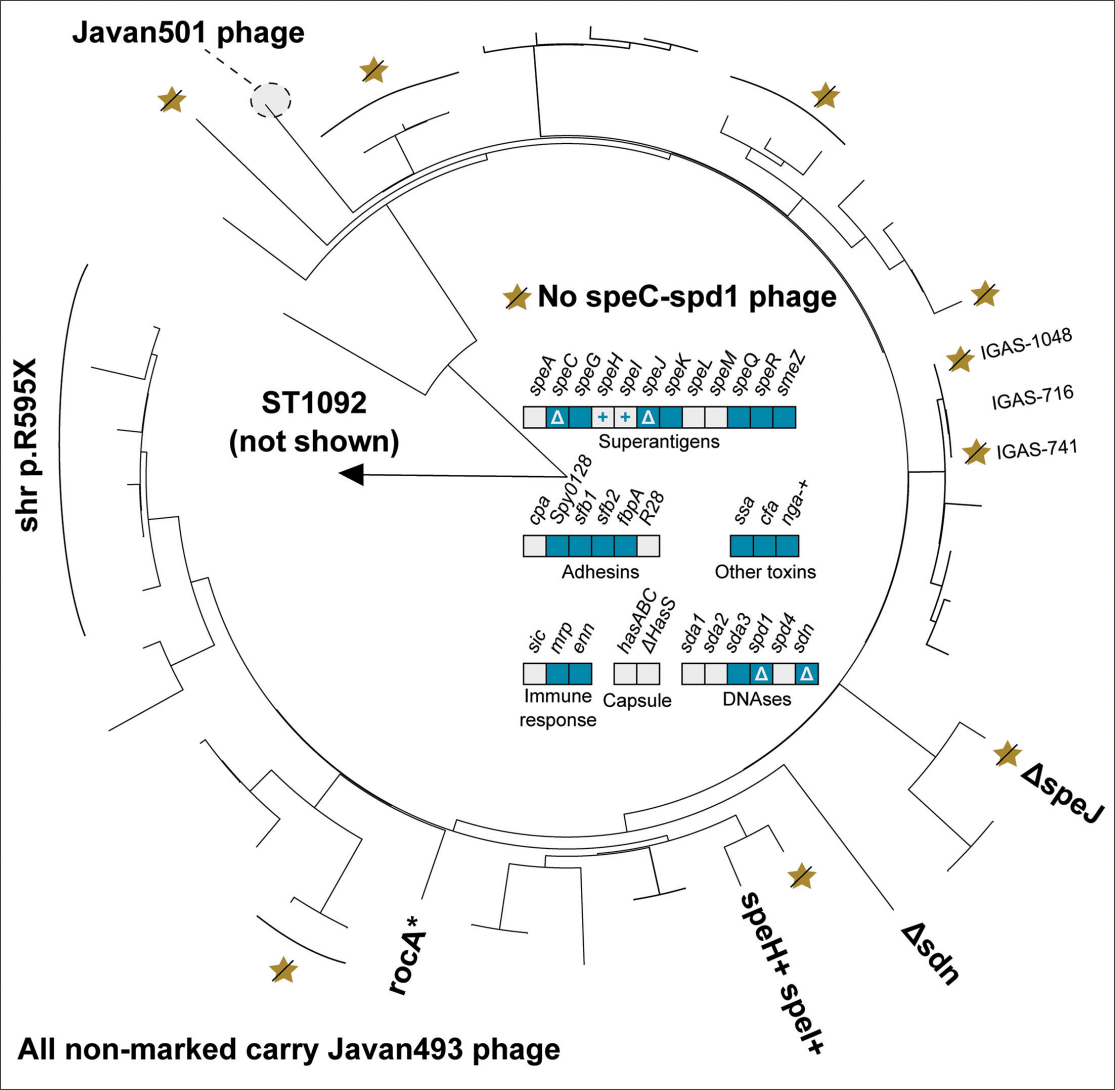
Phylogenetic tree of *emm*87 isolates from Norway, 2017−2023. Variations in accessory virulence factor content are annotated on branches, with lines perpendicular to the ring drawn to include multiple isolates where mutations are not just in singular isolates. A “+” indicates acquisition of a gene, and “Δ” a loss. The top right panel indicates the presence/absence of accessory virulence genes in *emm87*. Blue boxes indicate the presence and gray boxes indicate the absence of a virulence factor-encoding gene. A gray box with a “+” indicates sporadic gain, while a blue box with a “Δ” indicates sporadic loss. The majority of *emm87* isolates carried the *speC-spd1*-carrying phage Javan493, with one having switched to Javan501 and 14 having lost a *speC-spd1*-carrying phage (yellow star with line). Specific isolates referenced in the manuscript are named on the tree. A single ST1092 isolate, although categorized as *emm87.0*, was distantly related and not shown on the tree.

**Fig 7:**
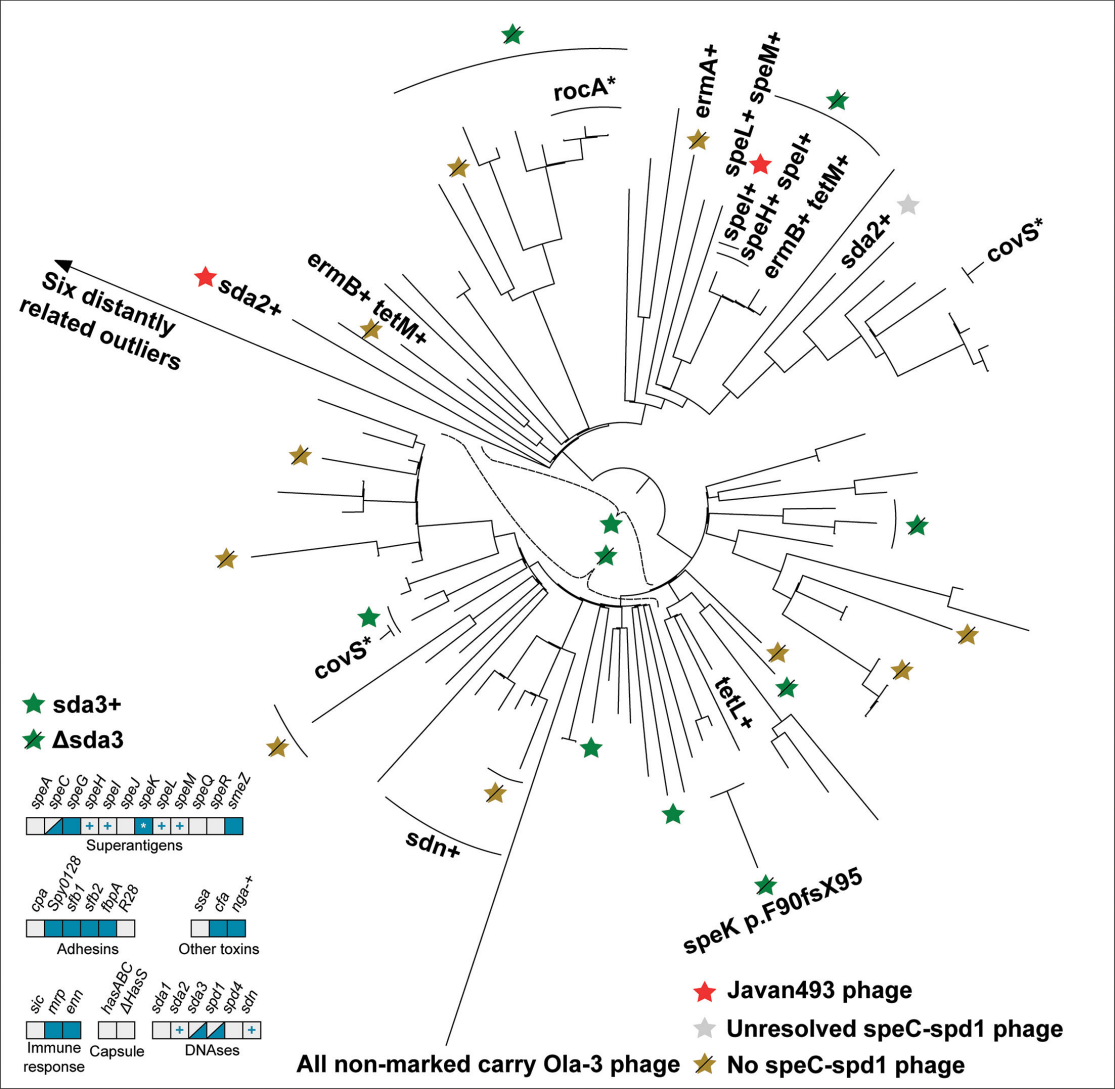
Phylogenetic tree of *emm*89 isolates from Norway, 2017−2023. Variations in accessory virulence factor content is annotated on branches, with lines perpendicular to the ring drawn to include multiple isolates where mutations are not just in singular isolates. A “+” indicates acquisition of a gene. The bottom left panel indicates the presence/absence of accessory virulence genes in *emm89*. Blue boxes indicate the presence and gray boxes indicate the absence of a virulence factor-encoding gene. Mixed blue and gray indicates variable presence. A gray box with a “+” indicates sporadic gain. A “*” indicates sporadic pseudogenization. Roughly 80% of the isolates had the *speC-spd1*-carrying Ola-3 phage, two had it switched with the Javan493 phage (red star), and approximately 15% had no *speC-spd1* phage (gray star). The presence of the Javan514 phage, carrying *sda3*, varied extensively throughout the phylogeny, as indicated by a green star (presence) and a green star with a line across (absence). The *speC-spd1*-carrying phage could not be resolved for one isolate. Six distantly related outliers are not shown on the tree.

**Fig 8:**
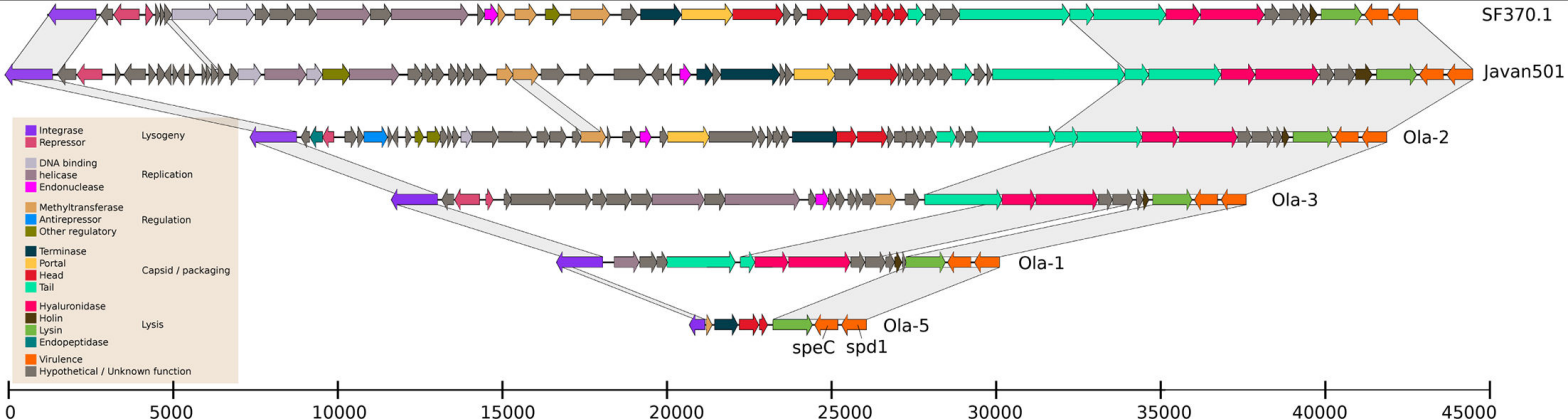
Genomic map of the structure of the various phages carrying virulence factors *speC* and *spd1*. The previously described SF370.1 and Javan501 are used as references on top. Open reading frames are colored by function. The virulence factor cargo genes *speC* and *spd1* are colored orange and to the right on all maps. Gray bars indicate significant BLAST homology (>80%) across the scrawled region, which is mostly restricted to the integrase as well as the phage tail, hyaluronidase, holin, and lysin parts of the phages, as well as the highly conserved *speC* and *spd1*.

In the following section, we describe the genomic characteristics of the six most common *emm* types currently circulating in Norway: *emm1, emm4, emm12, emm28, emm87,* and *emm89*. Each *emm* type had a distinct set of virulence factors, detailed in Table 3 as well as in Fig. 2 to 7. Chromosomal virulence factors were largely either present or absent within an *emm* type, while bacteriophage-associated virulence factors (such as most superantigens and DNAses) had a highly variable presence even within single *emm* types. This variation was caused by repeated infection and subsequent excision of a set of phages that carried a specific virulence factor cargo. Most notably, we found several different prophages encoding *speC* and *spd1* that had a highly variable pattern of presence within *emm1*, *emm12*, *emm87,* and *emm89*. All these phages had partial homology to each other and to SF370.1 in the integrase, tail, and lysis regions, but little homology across other regions (Fig. 8). We observed a pattern that strongly indicated repeated cycles of infection, lysogeny, and lytic release. For example, in *emm1*, isolates IGAS-608, IGAS-617, and IGAS-624 were nearly identical, with very short branch lengths between them (average branch length < 5.0E-7, roughly equivalent to a single nucleotide polymorphism over aligned regions) (Fig. 2). However, while IGAS-608 and IGAS-624 carried a *speC-spd1* phage that had >99% nucleotide identity over >96.5% of the length to Javan506 (MK448965), this phage was missing completely in IGAS-617, with the genes flanking the phage in IGAS-608 and IGAS-624 (*whiA* and *pepD*) being neighbors in IGAS-617. Similarly, in *emm87* (Fig. 6), IGAS-716 carried the Javan493 phage (>99% identity over >98% of the phage length) between *whiA* and *pepD*, but the phage was excised in the neighboring isolates IGAS-741 and IGAS-1048, both of which were related to IGAS-716 through branch lengths of approximately 1.0E-6, for example, roughly two polymorphisms over the aligned part of the genome. These observations point to ongoing conversion between lysogeny and lytic cycles at rapid intervals, certainly short enough that virtually no change in the vertically acquired part of the bacterial chromosome is detectable.

In general, phages would often be broken up into multiple contigs since the short Illumina reads do not span the phage’s repetitive elements. However, they were fully resolved in 70 genomes. The resolved *speC-spd1* phage sequences had a length between 5.9 and 45.9 kbp and were always integrated between *pepD*, a C69 family peptidase (WP_029714004), and the *whiA* gene (WP_002985428). Considering phages with >95% sequence identity as the same, the *speC-spd1* phages could be grouped into 11 distinctly different but related variants. Seven of these have been previously published (63): Javan501 (MK448782), Javan448 (MK448942), Javan460 (MK448949), Javan506 (MK448965), Javan516 (MK448970), Javan526 (MK448975), and Javan493 (MK448778). In addition, four previously undescribed phages were resolved, all of which display local sequence homology to the previously described phages, as well as to SF370.1, over the integrase and right part, including tail, hyaluronidase, lysin, and *speC-spd1*, but with low or absent homology at the protein level in other parts (Fig. 8). These have been designated Ola-1, Ola-2, Ola-3, and Ola-5. All pairwise comparisons between these four new phages show less than 70% intergenomic similarity, which suggests that they each represent new phage genera. Among the isolates belonging to the six major *emm* types, 474/845 (56.0%) carried one of the 11 related phages. These *speC-spd1*-encoding phages were mutually exclusive, with no isolates carrying more than one of these phages. This is most likely due to phage competition or superimmunity mediated through integrase homology and competition for the integration site between *whiA* and *pepD*. However, we observed several phage switches. For example, all *emm4* isolates carried one of the phages, but four different phage variants were found across the phylogeny of *emm4* (Fig. 3). A breakdown of different *speC-spd1*-carrying phages by *emm* type can be found in Table S2. Although phage defense systems vary by *emm* type (Table S3), we could not establish an obvious link between these and differential phage infection patterns.

Several other phages, seemingly non-competitive to the *speC-spd1* phages, were also found, but most were incompletely resolved by short-read data. One such phage, or several different ones, carried the *sdn* gene that encodes Streptodornase A, and these phages variably infected isolates within *emm4*, *emm12*, *emm28*, *emm87,* and *emm89*.

Similarly, a phage with > 99.9% sequence identity to the *sda3*-carrying phage Javan514 (MK448969) was present in 47.2% of *emm89* isolates (Fig. 7). The presence pattern of this phage also indicated repeated cycles of infection and excision. The minimum genomic distance between a phage-carrying and a non-infected isolate was 1.0E-5, equivalent to around 20 nucleotide differences. Across the *emm89* phylogenetic tree, the most parsimonious scenario implies four separate infections and six independent excision events of this phage. Several phages carrying *sda2* were also identified, but not fully resolved in *emm1*, *emm12*, *emm28,* and *emm89*.

We also identified the *speHI*-encoding prophage Φ9429.2 (NC_008021) in 98.2% of *emm28* isolates. A different but unresolved *speHI*-encoding phage occasionally infected *emm28* (1.0%), *emm87* (1.9%), and *emm89* (1.9%) isolates.

Other phage-associated virulence factors appeared to be more stable. For example, *emm12* could be divided into a *ssa*-positive and a *ssa*-negative clade, and within these clades, the presence or absence of the phage was constant. We could not resolve the *ssa*-carrying phage, but *ssa* was not carried on the same phage as *speC* and *spd1*, as is known to be the case in ΦHKU.vir (64).

Not all highly variable loci were phage-associated though. We did, for example, observe many instances of mutations that render strains incapable of producing capsule, either through truncations in the *hasABC* cluster (65), or HasS terminator mutations (57). Finally, sporadic *covS* mutations, known to be associated with increased pathogenicity (18), were seen in *emm1*, *emm4*, *emm12*, *emm28,* and *emm89*.

## DISCUSSION

Little is known about the genetic underpinnings of the many different clinical manifestations of invasive GAS infection. A European study across 11 countries found that STSS and NSTI were associated with *emm*1 and *emm*3 (66), and a large Spanish study found that *emm*4, *emm*12, and *emm*87 were associated with pharyngitis; *emm*1 and *emm*6 with otitis; *emm*89 with skin infections; and *emm*77 with genital infections (67). However, other studies found no associations at all (68). Our study unfortunately did not have access to clinical data, and we are therefore unable to evaluate the significance of specific virulence factors to clinical manifestations.

The relative frequency of *emm* types in our study was broadly comparable to that in other recent European studies (69, 70). The most common *emm* type was *emm1,* of which a majority (62.4%) were the hypervirulent M1_UK_ type. There was a significant relative increase in M1_UK_ during the study period, and it is noteworthy how quickly this variant has become dominant in Norway after its presumed emergence in the UK in 2008 (11). The pandemic period did impact this, with *emm1* becoming relatively less common in 2020−2021, likely because of the wide-reaching efforts to suppress Covid-19 infections during this period, including restrictions on international travel. On the other hand, the pandemic saw a significant increase in the relative importance of *emm89*, which could suggest more domestic patterns of transmission for *emm89* than for *emm1*. The pandemic years did not exhibit any seasonality in iGAS incidence, whereas in all other years, there was a strong pattern of infections peaking during Q1 and Q4. The post-pandemic years (2022−2023) saw a massive increase in iGAS incidence, and especially of *emm1* and *emm12*. It is unclear if this is due to the circulation of more virulent variants or a more general rebound effect, as this has been observed for many other infectious diseases post-COVID (71).

Treatment of resistant iGAS is a growing concern, both in Norway and the rest of the world, and reports indicate that resistance levels are over 90% in China (72). In this study, 15.8% of isolates were resistant to one or more antibiotics, and 6.1% to two or more. All isolates were susceptible to penicillin. Despite the relatively high level of tetracycline resistance, it should be noted that tetracycline is not used for the treatment of iGAS infections in Norway. There were no statistically significant changes in AMR levels throughout the study period, although the relative fraction of resistant types was higher during the pandemic years 2020−2021. Genotypic resistance testing was found to work well for tetracycline, clindamycin, and erythromycin but inadequately for trimethoprim-sulfamethoxazole.

Intriguingly, all major *emm* types had a pattern of repeated gain and subsequent loss of phage-associated virulence factors, notably the phage-associated superantigens (*speA*, *speC*, *speH*, *speI*, *speK*, *speL*, *speM*, and *ssa*) and DNAses (*sda1*, *sda2*, *sda3*, *spd1*, *spd4*, and *sdn*). This points to repeated cycles of lysogenic conversion and lytic escape of temperate phages, a dynamic presumably triggered by evolutionary trade-offs from different selective pressures in various environments and stress conditions (73). Several related variants of a ΦSF370.1-like phage carrying *speC* and *spd1* but not *ssa* were found to be repeatedly gained and lost across individual *emm* type phylogenies. Similar phages have been seen before (74), and variants of this phage have previously been reported to have a similar distribution pattern across *emm12* (64, 74), but our results suggest that phages carrying *speC* and *spd1* are more common and dynamic across different *emm* types than previously thought. It has been shown that human pharyngeal cells produce soluble factors that promote the induction of *speC*-carrying phages (75), but the frequency of subsequent reintegration makes the phage presence appear as potentially advantageous for the bacterium, for example, through superimmunity, the presence of the virulence factors themselves, or through modulation of transcription (76). Interestingly, the newly found phages were in various states of decay, as has been reported for SF370.1-family phages before (77). It seems likely that the highly variable presence of these virulence factors has an impact on bacterial pathogenicity beyond the already established link with scarlet fever, although further research is needed to more completely elucidate their role in systemic invasion and disease progression.

## MATERIALS AND METHODS

### Isolates

All suspected cases of iGAS occurring within Norway (including dependencies) are required by law to be reported through the Norwegian Surveillance System for Infectious Diseases (MSIS), and all culture-positive isolates should be submitted to the National Reference Laboratory, hosted at the Norwegian Institute of Public Health (NIPH). In this study, we analyzed the genomes of all isolates received at the National Reference Laboratory between 1 January 2017 and 30 April 2023 (1,218 in total). Several isolates were recovered from the same patient, from the same or different anatomical sites. To remove this pseudo-replication bias, only the last isolate was kept in the analyses when more than one isolate with the same *emm* type was obtained from an individual within a 30-day period. This removed 40 isolates. In addition, 14 isolates were discarded because of sub-optimal sequence quality or indications of contamination. Thus, the total number of included isolates was 1,163.

### Antimicrobial resistance and susceptibility testing in Norway

Antimicrobial susceptibility testing (AST) was performed by determining the minimum inhibitory concentration using the antimicrobial gradient strip diffusion method (AB Biodisk, Solna, Sweden). Briefly, isolates were grown overnight at 35°C with 5% CO_2_. Bacteria were suspended in Mueller-Hinton broth to a density of 0.5 McFarland and plated on blood agar plates, after which antibiotic strips were overlaid. The plates were then incubated overnight at 35°C with 5% CO_2_ , and the MICs were read the following day. Antibiotics tested were penicillin G, erythromycin, clindamycin, tetracycline, and trimethoprim-sulfamethoxazole (23). MIC breakpoint values were taken from the European Committee on Antimicrobial Susceptibility Testing (EUCAST) Clinical Breakpoint Table v. 14.0.

For budgetary reasons, AST was not routinely performed between March 2018 and December 2019. This means that only 854 out of 1,163 (73.4%) isolates had associated ASTs. For validation, all isolates from this period that carried genetic resistance markers were retrospectively cultured for AST, and indeed, all these 47 isolates displayed phenotypic resistance. However, since this represents a form of selection bias, we did not include these 47 isolates when calculating correlations between genotypic and phenotypic resistance.

### Whole genome sequencing

The isolates were grown overnight on Columbia agar with 5% defibrinated horse blood in 5% CO_2_ at 35°C for DNA extraction. DNA was extracted using MagNA Pure 96 (Roche Life Science), and DNA sequencing libraries were prepared using KAPA HyperPlus kits (Roche Life Science) with NEXTflex DNA barcodes (Bio Scientific) following the manufacturer’s instructions. The DNA libraries were sequenced on a MiSeq or NextSeq platform (Illumina) using the v2 500-cycles or the v3 600 cycles reagents kits (Illumina), following the manufacturer’s instructions.

### Genome analyses

Raw Illumina paired-end reads were trimmed using fastp v0.23.4 (78). Quality assessment of the trimmed reads and assembled genomes was performed using FastQC v0.12.1 (http://bioinformatics.babraham.ac.uk/projects/fastqc), QUAST v5.2.0 (79), and MultiQC v1.21 (80). *De novo* genome assembly was carried out using Unicycler v0.4.8 (81, 82), and the genomes were annotated using Prokka 1.14.6 (83). *In silico emm* typing and *emm* clustering (84) were done using emmtyper v0.2.0 (https://github.com/MDU-PHL/emmtyper). Genotypic antimicrobial resistance was predicted using StarAMR v0.10.0 (85). Gene clustering and alignment were done with Panaroo v1.5.0 (86). Extraction and typing of specific genes were done manually using BLASTN v2.12.0+ of a set of reference genes (Table 3: Overview of virulence factors) against all open reading frames. Promoters and terminators were extracted using SeqKit v2.8.2 (87). Phage characterization was done using Phastest (88), and phage homology was assessed with BLASTN. Phage defense systems were characterized with DefenseFinder v2.0.1 (89, 90).

Recombination-free sequence alignments were created using the distance tree approach implemented in Verticall (91). Phylogenetic trees were constructed using IQ-TREE v2.3.3 (92–94), using a Kimura 3-parameter with fixed and invariant sites, as determined by ModelFinder. Phylogenetic trees were annotated in iTol v6 (95).

### Descriptive statistical analyses

Samples were categorized by main *emm* types (1, 4, 12, 53, 55, 66) or grouped into ”Other,” representing the remaining 55 *emm* types that each appear 50 times or less in our data set. Univariate logistic regression was used to identify factors with significantly higher or lower occurrences of a given *emm* type compared to all other factor levels using the ‘caret’ package (version 6.0-94) in R v4.4.0. The geographic factors were East, South, West, Mid, and Northern Norway. Patient age was grouped into 0−9 years, 10−19 years, 20−39 years, 40−69 years, and 70+ years. Furthermore, we included the dichotomous variables sex (female vs. male), season (winter: October−March, summer: April−September), and whether the isolate was from the pandemic period (samples collected between March 14, 2020 and February 1, 2021). False discovery rate (FDR)-adjusted *P*-values were calculated to account for multiple comparisons.

## Data Availability

Sequence data for all isolates included in this study have been uploaded to the European Nucleotide Archive (ENA) under accession number PRJEB108928. All supporting data, code, and protocols have been provided within the article and its supplemental material.

## SUPPLEMENTAL MATERIAL

Fig. S1 msphere.00775-25-s0001.eps Annual incidence rates.

Fig. S2 msphere.00775-25-s0002.eps Phylogenetic tree of all 1,163 isolates included in this study.

Legends msphere.00775-25-s0003.docx Legends for supplemental material.

Table S1 msphere.00775-25-s0004.xlsx Metadata.

Table S2 msphere.00775-25-s0005.docx Phage presence by *emm* type.

Table S3 msphere.00775-25-s0006.docx Phage defense systems.

ASM does not own the copyrights to Supplemental Material that may be linked to, or accessed through, an article. The authors have granted ASM a non-exclusive, world-wide license to publish the Supplemental Material files. Please contact the corresponding author directly for reuse.

## References

[1] Kanwal S, Vaitla P. 2021. StatPearls [Internet]. Streptococcus pyogenes. StatPearls Publishing, Treasure Island (FL).

[2] Brouwer S, Rivera-Hernandez T, Curren BF, Harbison-Price N, De Oliveira DMP, Jespersen MG, Davies MR, Walker MJ. 2023. Pathogenesis, epidemiology and control of group A Streptococcus infection. Nat Rev Microbiol 21:431–447. doi:10.1038/s41579-023-00865-7 36894668 PMC9998027

[3] Smeesters PR, de Crombrugghe G, Tsoi SK, Leclercq C, Baker C, Osowicki J, Verhoeven C, Botteaux A, Steer AC. 2024. Global Streptococcus pyogenes strain diversity, disease associations, and implications for vaccine development: a systematic review. Lancet Microbe 5:e181–e193. doi:10.1016/S2666-5247(23)00318-X 38070538

[4] Su Y-F, Wang S-M, Lin Y-L, Chuang W-J, Lin Y-S, Wu J-J, Lin MT, Liu C-C. 2009. Changing epidemiology of Streptococcus pyogenes emm types and associated invasive and noninvasive infections in Southern Taiwan. J Clin Microbiol 47:2658–2661. doi:10.1128/JCM.01078-09 19515840 PMC2725672

[5] Steer AC, Law I, Matatolu L, Beall BW, Carapetis JR. 2009. Global emm type distribution of group A streptococci: systematic review and implications for vaccine development. Lancet Infect Dis 9:611–616. doi:10.1016/S1473-3099(09)70178-1 19778763

[6] Lynskey NN, Jauneikaite E, Li HK, Zhi X, Turner CE, Mosavie M, Pearson M, Asai M, Lobkowicz L, Chow JY, Parkhill J, Lamagni T, Chalker VJ, Sriskandan S. 2019. Emergence of dominant toxigenic M1T1 Streptococcus pyogenes clone during increased scarlet fever activity in England: a population-based molecular epidemiological study. Lancet Infect Dis 19:1209–1218. doi:10.1016/S1473-3099(19)30446-3 31519541 PMC6838661

[7] Davies MR, Keller N, Brouwer S, Jespersen MG, Cork AJ, Hayes AJ, Pitt ME, De Oliveira DMP, Harbison-Price N, Bertolla OM, et al.. 2023. Detection of Streptococcus pyogenes M1_UK_ in Australia and characterization of the mutation driving enhanced expression of superantigen SpeA. Nat Commun 14:1051. doi:10.1038/s41467-023-36717-4 36828918 PMC9951164

[8] Demczuk W, Martin I, Domingo FR, MacDonald D, Mulvey MR. 2019. Identification of Streptococcus pyogenes M1UK clone in Canada. Lancet Infect Dis 19:1284–1285. doi:10.1016/S1473-3099(19)30622-X 31782392

[9] Gouveia C, Bajanca-Lavado MP, Mamede R, Carvalho AA, Rodrigues F, Melo-Cristino J. 2023. Sustained increase of paediatric invasive Streptococcus pyogenes infections dominated by M1UK and diverse emm12 isolates, Portugal, September 2022 to May 2023. Euro Surveill 28:2300427. doi:10.2807/1560-7917.ES.2023.28.36.2300427 37676143 PMC10486195

[10] Li Y, Nanduri SA, Van Beneden CA, Beall BW. 2020. M1_UK_ lineage in invasive group A Streptococcus isolates from the USA. Lancet Infect Dis 20:538–539. doi:10.1016/S1473-3099(20)30279-6 PMC9052193 32359463

[11] Vieira A, Wan Y, Ryan Y, Li HK, Guy RL, Papangeli M, Huse KK, Reeves LC, Soo VWC, Daniel R, et al.. 2024. Rapid expansion and international spread of M1_UK_ in the post-pandemic UK upsurge of Streptococcus pyogenes. Nat Commun 15:3916. doi:10.1038/s41467-024-47929-7 38729927 PMC11087535

[12] Bergsten H, Nizet V. 2024. The intricate pathogenicity of group A Streptococcus: A comprehensive update. Virulence 15:2412745. doi:10.1080/21505594.2024.2412745 39370779 PMC11542602

[13] Hynes W. 2004. Virulence factors of the group A streptococci and genes that regulate their expression. Front Biosci 9:3399–3433. doi:10.2741/1491 15353367

[14] Davies MR, McIntyre L, Mutreja A, Lacey JA, Lees JA, Towers RJ, Duchêne S, Smeesters PR, Frost HR, Price DJ, et al.. 2019. Atlas of group A streptococcal vaccine candidates compiled using large-scale comparative genomics. Nat Genet 51:1035–1043. doi:10.1038/s41588-019-0417-8 31133745 PMC6650292

[15] Walkinshaw DR, Wright MEE, Mullin AE, Excler J-L, Kim JH, Steer AC. 2023. The Streptococcus pyogenes vaccine landscape. NPJ Vaccines 8:16. doi:10.1038/s41541-023-00609-x 36788225 PMC9925938

[16] Schiavolin L, Deneubourg G, Steinmetz J, Smeesters PR, Botteaux A. 2024. Group A Streptococcus adaptation to diverse niches: lessons from transcriptomic studies. Crit Rev Microbiol 50:241–265. doi:10.1080/1040841X.2023.2294905 38140809

[17] Falaleeva M, Zurek OW, Watkins RL, Reed RW, Ali H, Sumby P, Voyich JM, Korotkova N. 2014. Transcription of the Streptococcus pyogenes hyaluronic acid capsule biosynthesis operon is regulated by previously unknown upstream elements. Infect Immun 82:5293–5307. doi:10.1128/IAI.02035-14 25287924 PMC4249290

[18] Tatsuno I, Okada R, Zhang Y, Isaka M, Hasegawa T. 2013. Partial loss of CovS function in Streptococcus pyogenes causes severe invasive disease. BMC Res Notes 6:126. doi:10.1186/1756-0500-6-126 23537349 PMC3637574

[19] Li Y, Dominguez S, Nanduri SA, Rivers J, Mathis S, Li Z, McGee L, Chochua S, Metcalf BJ, Van Beneden CA, Beall B, Miller L. 2022. Genomic characterization of group A streptococci causing pharyngitis and invasive disease in Colorado, USA, June 2016- April 2017. J Infect Dis 225:1841–1851. doi:10.1093/infdis/jiab565 34788828 PMC9125432

[20] Dickinson H, Reacher M, Nazareth B, Eagle H, Fowler D, Underwood A, Chand M, Chalker V, Coelho J, Daniel R, Kapatai G, Al-Shabib A, Puleston R. 2019. Whole-genome sequencing in the investigation of recurrent invasive group A Streptococcus outbreaks in a maternity unit. J Hosp Infect 101:320–326. doi:10.1016/j.jhin.2018.03.018 29577990

[21] van der Putten BCL, Bril-Keijzers WCM, Rumke LW, Vestjens SMT, Koster LAM, Willemsen M, van Houten MA, Rots NY, Vlaminckx BJM, de Gier B, van Sorge NM. 2023. Novel emm4 lineage associated with an upsurge in invasive group A streptococcal disease in the Netherlands, 2022. Microb Genom 9:mgen001026. doi:10.1099/mgen.0.001026 37261428 PMC10327499

[22] Haenni M, Lupo A, Madec J-Y. 2018. Antimicrobial resistance in Streptococcus spp. Microbiol Spectr 6:6. doi:10.1128/microbiolspec.ARBA-0008-2017 PMC11633561 29600772

[23] NORM/NORM-VET. 2023. Usage of antimicrobial agents and occurrence of antimicrobial resistance in Norway. Tromsø / Ås / Oslo

[24] Shannon BA, McCormick JK, Schlievert PM. 2019. Toxins and superantigens of group A streptococci. Microbiol Spectr 7:7. doi:10.1128/microbiolspec.gpp3-0054-2018 PMC11590448 30737912

[25] Reglinski M, Sriskandan S, Turner CE. 2019. Identification of two new core chromosome-encoded superantigens in Streptococcus pyogenes; speQ and speR. J Infect 78:358–363. doi:10.1016/j.jinf.2019.02.005 30796950

[26] Reda KB, Kapur V, Mollick JA, Lamphear JG, Musser JM, Rich RR. 1994. Molecular characterization and phylogenetic distribution of the streptococcal superantigen gene (ssa) from Streptococcus pyogenes. Infect Immun 62:1867–1874. doi:10.1128/iai.62.5.1867-1874.1994 8168951 PMC186429

[27] Hytönen J, Haataja S, Gerlach D, Podbielski A, Finne J. 2001. The SpeB virulence factor of Streptococcus pyogenes, a multifunctional secreted and cell surface molecule with strepadhesin, laminin-binding and cysteine protease activity. Mol Microbiol 39:512–519. doi:10.1046/j.1365-2958.2001.02269.x 11136470

[28] Langshaw EL, Reynolds S, Ozberk V, Dooley J, Calcutt A, Zaman M, Walker MJ, Batzloff MR, Davies MR, Good MF, Pandey M. 2023. Streptolysin O deficiency in Streptococcus pyogenes M1T1 covR/S mutant strain attenuates virulence in in vitro and in vivo infection models. mBio 14:e03488–22. doi:10.1128/mbio.03488-22 36744883 PMC9972915

[29] Molloy EM, Cotter PD, Hill C, Mitchell DA, Ross RP. 2011. Streptolysin S-like virulence factors: the continuing sagA. Nat Rev Microbiol 9:670–681. doi:10.1038/nrmicro2624 21822292 PMC3928602

[30] Barnett TC, Cole JN, Rivera-Hernandez T, Henningham A, Paton JC, Nizet V, Walker MJ. 2015. Streptococcal toxins: role in pathogenesis and disease: streptococcal toxins role in pathogenesis and disease. Cell Microbiol 17:1721–1741. doi:10.1111/cmi.12531 26433203

[31] Smith CL, Ghosh J, Elam JS, Pinkner JS, Hultgren SJ, Caparon MG, Ellenberger T. 2011. Structural basis of Streptococcus pyogenes immunity to its NAD+ glycohydrolase toxin. Structure 19:192–202. doi:10.1016/j.str.2010.12.013 21300288 PMC3056158

[32] Gase K, Ferretti JJ, Primeaux C, McShan WM. 1999. Identification, cloning, and expression of the CAMP factor gene (cfa) of group A streptococci. Infect Immun 67:4725–4731. doi:10.1128/IAI.67.9.4725-4731.1999 10456923 PMC96801

[33] Ashbaugh CD, Albertí S, Wessels MR. 1998. Molecular analysis of the capsule gene region of group A Streptococcus: the hasAB genes are sufficient for capsule expression. J Bacteriol 180:4955–4959. doi:10.1128/JB.180.18.4955-4959.1998 9733702 PMC107524

[34] Hynes WL, Walton SL. 2000. Hyaluronidases of gram-positive bacteria. FEMS Microbiol Lett 183:201–207. doi:10.1111/j.1574-6968.2000.tb08958.x 10675584

[35] Kreikemeyer B, Nakata M, Oehmcke S, Gschwendtner C, Normann J, Podbielski A. 2005. Streptococcus pyogenes collagen type I-binding Cpa surface protein. Journal of Biological Chemistry 280:33228–33239. doi:10.1074/jbc.M502896200 16040603

[36] Abbot EL, Smith WD, Siou GPS, Chiriboga C, Smith RJ, Wilson JA, Hirst BH, Kehoe MA. 2007. Pili mediate specific adhesion of Streptococcus pyogenes to human tonsil and skin. Cell Microbiol 9:1822–1833. doi:10.1111/j.1462-5822.2007.00918.x 17359232

[37] Yamaguchi M, Terao Y, Kawabata S. 2013. Pleiotropic virulence factor - Streptococcus pyogenes fibronectin-binding proteins: Multiple role of S. pyogenes Fn-binding proteins. Cell Microbiol 15:503–511. doi:10.1111/cmi.12083 23190012

[38] Lukomski S, Bachert BA, Squeglia F, Berisio R. 2017. Collagen-like proteins of pathogenic streptococci. Mol Microbiol 103:919–930. doi:10.1111/mmi.13604 27997716 PMC5344740

[39] Eraso JM, Kachroo P, Olsen RJ, Beres SB, Zhu L, Badu T, Shannon S, Cantu CC, Saavedra MO, Kubiak SL, Porter AR, DeLeo FR, Musser JM. 2020. Genetic heterogeneity of the Spy1336/R28-Spy1337 virulence axis in Streptococcus pyogenes and effect on gene transcript levels and pathogenesis. PLoS One 15:e0229064. doi:10.1371/journal.pone.0229064 32214338 PMC7098570

[40] Ayinuola YA, Tjia-Fleck S, Readnour BM, Liang Z, Ayinuola O, Paul LN, Lee SW, Fischetti VA, Ploplis VA, Castellino FJ. 2022. Relationships between plasminogen-binding M-protein and surface enolase for human plasminogen acquisition and activation in Streptococcus pyogenes. Front Microbiol 13:905670. doi:10.3389/fmicb.2022.905670 35685926 PMC9173704

[41] Akesson P, Sjöholm AG, Björck L. 1996. Protein SIC, a novel extracellular protein of Streptococcus pyogenes interfering with complement function. J Biol Chem 271:1081–1088. doi:10.1074/jbc.271.2.1081 8557634

[42] Nitzsche R, Rosenheinrich M, Kreikemeyer B, Oehmcke-Hecht S. 2015. Streptococcus pyogenes triggers activation of the human contact system by streptokinase. Infect Immun 83:3035–3042. doi:10.1128/IAI.00180-15 25987706 PMC4496597

[43] Ellen RP, Gibbons RJ. 1972. M protein-associated adherence of Streptococcus pyogenes to epithelial surfaces: prerequisite for virulence. Infect Immun 5:826–830. doi:10.1128/iai.5.5.826-830.1972 4564883 PMC422446

[44] Podbielski A, Schnitzler N, Beyhs P, Boyle MDP. 1996. M-related protein (Mrp) contributes to group A streptococcal resistance to phagocytosis by human granulocytes. Mol Microbiol 19:429–441. doi:10.1046/j.1365-2958.1996.377910.x 8830235

[45] Proctor E-J, Frost HR, Satapathy S, Botquin G, Urbaniec J, Gorman J, De Oliveira DMP, McArthur J, Davies MR, Botteaux A, Smeesters P, Sanderson-Smith M. 2024. Molecular characterization of the interaction between human IgG and the M-related proteins from Streptococcus pyogenes. J Biol Chem 300:105623. doi:10.1016/j.jbc.2023.105623 38176650 PMC10844976

[46] Wierzbicki IH, Campeau A, Dehaini D, Holay M, Wei X, Greene T, Ying M, Sands JS, Lamsa A, Zuniga E, Pogliano K, Fang RH, LaRock CN, Zhang L, Gonzalez DJ. 2019. Group A streptococcal S protein utilizes red blood cells as immune camouflage and is a critical determinant for immune evasion. Cell Rep 29:2979–2989. doi:10.1016/j.celrep.2019.11.001 31801066 PMC6951797

[47] Turner CE, Kurupati P, Jones MD, Edwards RJ, Sriskandan S. 2009. Emerging role of the interleukin-8 cleaving enzyme SpyCEP in clinical Streptococcus pyogenes infection. J Infect Dis 200:555–563. doi:10.1086/603541 19591574 PMC2820315

[48] Chen CC, Cleary PP. 1990. Complete nucleotide sequence of the streptococcal C5a peptidase gene of Streptococcus pyogenes. J Biol Chem 265:3161–3167. doi:10.1016/S0021-9258(19)39748-0 2406246

[49] Söderberg JJ, Engström P, von Pawel-Rammingen U. 2008. The intrinsic immunoglobulin G endopeptidase activity of streptococcal Mac-2 proteins implies a unique role for the enzymatically impaired Mac-2 protein of M28 serotype strains. Infect Immun 76:2183–2188. doi:10.1128/IAI.01422-07 18332209 PMC2346711

[50] Trastoy B, Lomino JV, Pierce BG, Carter LG, Günther S, Giddens JP, Snyder GA, Weiss TM, Weng Z, Wang L-X, Sundberg EJ. 2014. Crystal structure of Streptococcus pyogenes EndoS, an immunomodulatory endoglycosidase specific for human IgG antibodies. Proc Natl Acad Sci USA 111:6714–6719. doi:10.1073/pnas.1322908111 24753590 PMC4020096

[51] Remmington A, Turner CE. 2018. The DNases of pathogenic Lancefield streptococci. Microbiology (Reading) 164:242–250. doi:10.1099/mic.0.000612 29458565

[52] Kreikemeyer B, McIver KS, Podbielski A. 2003. Virulence factor regulation and regulatory networks in Streptococcus pyogenes and their impact on pathogen-host interactions. Trends Microbiol 11:224–232. doi:10.1016/s0966-842x(03)00098-2 12781526

[53] Biswas I, Scott JR. 2003. Identification of rocA, a positive regulator of covR expression in the group A Streptococcus. J Bacteriol 185:3081–3090. doi:10.1128/JB.185.10.3081-3090.2003 12730168 PMC154078

[54] Lambert C, Gaillard M, Wongdontree P, Bachmann C, Hautcoeur A, Gloux K, Guilbert T, Méhats C, Prost B, Solgadi A, Abreu S, Andrieu M, et al.. 2024. The double-edged role of FASII regulator FabT in Streptococcus pyogenes infection. Nat Commun 15:8593. doi:10.1038/s41467-024-52637-3 39366941 PMC11452403

[55] Bates CS, Toukoki C, Neely MN, Eichenbaum Z. 2005. Characterization of MtsR, a new metal regulator in group A Streptococcus, involved in iron acquisition and virulence. Infect Immun 73:5743–5753. doi:10.1128/IAI.73.9.5743-5753.2005 16113291 PMC1231137

[56] Leday TV, Gold KM, Kinkel TL, Roberts SA, Scott JR, McIver KS. 2008. TrxR, a new CovR-repressed response regulator that activates the Mga virulence regulon in group A Streptococcus. Infect Immun 76:4659–4668. doi:10.1128/IAI.00597-08 18678666 PMC2546847

[57] Turner CE, Holden MTG, Blane B, Horner C, Peacock SJ, Sriskandan S. 2019. The emergence of successful Streptococcus pyogenes lineages through convergent pathways of capsule loss and recombination directing high toxin expression. mBio 10:10–1128. doi:10.1128/mBio.02521-19 PMC6904876 31822586

[58] Le Rhun A, Lécrivain A-L, Reimegård J, Proux-Wéra E, Broglia L, Della Beffa C, Charpentier E. 2017. Identification of endoribonuclease specific cleavage positions reveals novel targets of RNase III in Streptococcus pyogenes. Nucleic Acids Res 45:2329–2340. doi:10.1093/nar/gkw1316 28082390 PMC5389636

[59] Chen Z, Itzek A, Malke H, Ferretti JJ, Kreth J. 2013. Multiple roles of RNase Y in Streptococcus pyogenes mRNA processing and degradation. J Bacteriol 195:2585–2594. doi:10.1128/JB.00097-13 23543715 PMC3676074

[60] Lécrivain A-L, Le Rhun A, Renault TT, Ahmed-Begrich R, Hahnke K, Charpentier E. 2018. In vivo 3′-to-5′ exoribonuclease targetomes of Streptococcus pyogenes. Proc Natl Acad Sci USA 115:11814–11819. doi:10.1073/pnas.1809663115 30381461 PMC6243249

[61] DebRoy S, Shropshire WC, Tran CN, Hao H, Gohel M, Galloway-Peña J, Hanson B, Flores AR, Shelburne SA. 2021. Characterization of the type I restriction modification system broadly conserved among group A streptococci. mSphere 6:e0079921. doi:10.1128/mSphere.00799-21 34787444 PMC8597746

[62] Dahesh S, Nizet V, Cole JN. 2012. Study of streptococcal hemoprotein receptor (Shr) in iron acquisition and virulence of M1T1 group A Streptococcus. Virulence 3:566–575. doi:10.4161/viru.21933 23076332 PMC3545933

[63] Rezaei Javan R, Ramos-Sevillano E, Akter A, Brown J, Brueggemann AB. 2019. Prophages and satellite prophages are widespread in Streptococcus and may play a role in pneumococcal pathogenesis. Nat Commun 10:4852. doi:10.1038/s41467-019-12825-y 31649284 PMC6813308

[64] Davies MR, Holden MT, Coupland P, Chen JHK, Venturini C, Barnett TC, Zakour NLB, Tse H, Dougan G, Yuen K-Y, Walker MJ. 2015. Emergence of scarlet fever Streptococcus pyogenes emm12 clones in Hong Kong is associated with toxin acquisition and multidrug resistance. Nat Genet 47:84–87. doi:10.1038/ng.3147 25401300

[65] Hall JN, Bah SY, Khalid H, Brailey A, Coleman S, Kirk T, Hussain N, Tovey M, Chaudhuri RR, Davies S, Tilley L, de Silva T, Turner CE. 2024. Molecular characterization of Streptococcus pyogenes (StrepA) non-invasive isolates during the 2022-2023 UK upsurge. Microb Genom 10:001277. doi:10.1099/mgen.0.001277 39133528 PMC11318961

[66] Luca-Harari B, Darenberg J, Neal S, Siljander T, Strakova L, Tanna A, Creti R, Ekelund K, Koliou M, van der Linden M, Henriques-Normark B, Jasir A, et al.. 2009. Clinical and microbiological characteristics of severe Streptococcus pyogenes disease in Europe. J Clin Microbiol 47:1155–1165. doi:10.1128/JCM.02155-08 19158266 PMC2668334

[67] Tamayo E, Montes M, García-Arenzana JM, Pérez-Trallero E. 2014. Streptococcus pyogenes emm-types in northern Spain; population dynamics over a 7-year period. J Infect 68:50–57. doi:10.1016/j.jinf.2013.08.013 23999149

[68] Rantala S, Vähäkuopus S, Siljander T, Vuopio J, Huhtala H, Vuento R, Syrjänen J. 2012. Streptococcus pyogenes bacteraemia, emm types and superantigen profiles. Eur J Clin Microbiol Infect Dis 31:859–865. doi:10.1007/s10096-011-1385-9 21877175

[69] Ramírez de Arellano E, Saavedra-Lozano J, Villalón P, Jové-Blanco A, Grandioso D, Sotelo J, Gamell A, González-López JJ, Cervantes E, Gónzalez MJ, et al.. 2024. Clinical, microbiological, and molecular characterization of pediatric invasive infections by Streptococcus pyogenes in Spain in a context of global outbreak. mSphere 9:e0072923. doi:10.1128/msphere.00729-23 38440985 PMC10964401

[70] Goldberg-Bockhorn E, Hagemann B, Furitsch M, Hoffmann TK. 2024. Invasive group A streptococcal infections in Europe after the COVID-19 pandemic. Dtsch Arztebl Int 121:673–680. doi:10.3238/arztebl.m2024.0127 38961826 PMC11966131

[71] Feinmann J. 2024. Analysis reveals global post-covid surge in infectious diseases. BMJ:q1348. doi:10.1136/bmj.q1348 38889948

[72] Lu B, Fang Y, Fan Y, Chen X, Wang J, Zeng J, Li Y, Zhang Z, Huang L, Li H, Li D, Zhu F, Cui Y, Wang D. 2017. High prevalence of macrolide-resistance and molecular characterization of Streptococcus pyogenes isolates circulating in China from 2009 to 2016. Front Microbiol 8:1052. doi:10.3389/fmicb.2017.01052 28642756 PMC5463034

[73] Zhang M, Zhang T, Yu M, Chen YL, Jin M. 2022. The life cycle transitions of temperate phages: regulating factors and potential ecological implications. Viruses 14:1904. doi:10.3390/v14091904 36146712 PMC9502458

[74] Tse H, Bao JYJ, Davies MR, Maamary P, Tsoi H-W, Tong AHY, Ho TCC, Lin C-H, Gillen CM, Barnett TC, Chen JHK, et al.. 2012. Molecular characterization of the 2011 Hong Kong scarlet fever outbreak. J Infect Dis 206:341–351. doi:10.1093/infdis/jis362 22615319 PMC4125623

[75] Broudy TB, Pancholi V, Fischetti VA. 2001. Induction of lysogenic bacteriophage and phage-associated toxin from group a streptococci during coculture with human pharyngeal cells. Infect Immun 69:1440–1443. doi:10.1128/IAI.69.3.1440-1443.2001 11179310 PMC98039

[76] McShan WM, McCullor KA, Nguyen SV. 2019. The bacteriophages of Streptococcus pyogenes. Microbiol Spectr 7:10–1128. doi:10.1128/microbiolspec.gpp3-0059-2018 PMC11314938 31111820

[77] Canchaya C, Desiere F, McShan WM, Ferretti JJ, Parkhill J, Brüssow H. 2002. Genome analysis of an inducible prophage and prophage remnants integrated in the Streptococcus pyogenes strain SF370. Virology (Auckl) 302:245–258. doi:10.1006/viro.2002.1570 12441069

[78] Chen S, Zhou Y, Chen Y, Gu J. 2018. Fastp: an ultra-fast all-in-one FASTQ preprocessor. Bioinformatics 34:i884–i890. doi:10.1093/bioinformatics/bty560 30423086 PMC6129281

[79] Gurevich A, Saveliev V, Vyahhi N, Tesler G. 2013. QUAST: quality assessment tool for genome assemblies. Bioinformatics 29:1072–1075. doi:10.1093/bioinformatics/btt086 23422339 PMC3624806

[80] Ewels P, Magnusson M, Lundin S, Käller M. 2016. MultiQC: summarize analysis results for multiple tools and samples in a single report. Bioinformatics 32:3047–3048. doi:10.1093/bioinformatics/btw354 27312411 PMC5039924

[81] Wick RR, Judd LM, Gorrie CL, Holt KE. 2017. Unicycler: resolving bacterial genome assemblies from short and long sequencing reads. PLoS Comput Biol 13:e1005595. doi:10.1371/journal.pcbi.1005595 28594827 PMC5481147

[82] Bankevich A, Nurk S, Antipov D, Gurevich AA, Dvorkin M, Kulikov AS, Lesin VM, Nikolenko SI, Pham S, Prjibelski AD, Pyshkin AV, Sirotkin AV, et al.. 2012. SPAdes: a new genome assembly algorithm and its applications to single-cell sequencing. J Comput Biol 19:455–477. doi:10.1089/cmb.2012.0021 22506599 PMC3342519

[83] Seemann T. 2014. Prokka: rapid prokaryotic genome annotation. Bioinformatics 30:2068–2069. doi:10.1093/bioinformatics/btu153 24642063

[84] Sanderson-Smith M, De Oliveira DMP, Guglielmini J, McMillan DJ, Vu T, Holien JK, Henningham A, Steer AC, Bessen DE, Dale JB, Curtis N, Beall BW, et al.. 2014. A systematic and functional classification of Streptococcus pyogenes that serves as a new tool for molecular typing and vaccine development. J Infect Dis 210:1325–1338. doi:10.1093/infdis/jiu260 24799598 PMC6083926

[85] Bharat A, Petkau A, Avery BP, Chen JC, Folster JP, Carson CA, Kearney A, Nadon C, Mabon P, Thiessen J, Alexander DC, Allen V, El Bailey S, Bekal S, German GJ, Haldane D, Hoang L, Chui L, Minion J, Zahariadis G, Domselaar GV, Reid-Smith RJ, Mulvey MR. 2022. Correlation between phenotypic and in silico detection of antimicrobial resistance in Salmonella enterica in Canada using Staramr. Microorganisms 10:292. doi:10.3390/microorganisms10020292 35208747 PMC8875511

[86] Tonkin-Hill G, MacAlasdair N, Ruis C, Weimann A, Horesh G, Lees JA, Gladstone RA, Lo S, Beaudoin C, Floto RA, Frost SDW, Corander J, et al.. 2020. Producing polished prokaryotic pangenomes with the Panaroo pipeline. Genome Biol 21:180. doi:10.1186/s13059-020-02090-4 32698896 PMC7376924

[87] Shen W, Sipos B, Zhao L. 2024. SeqKit2: a swiss army knife for sequence and alignment processing. Imeta 3:e191. doi:10.1002/imt2.191 38898985 PMC11183193

[88] Wishart DS, Han S, Saha S, Oler E, Peters H, Grant JR, Stothard P, Gautam V. 2023. PHASTEST: faster than PHASTER, better than PHAST. Nucleic Acids Res 51:W443–W450. doi:10.1093/nar/gkad382 37194694 PMC10320120

[89] Tesson F, Hervé A, Mordret E, Touchon M, d’Humières C, Cury J, Bernheim A. 2022. Systematic and quantitative view of the antiviral arsenal of prokaryotes. Nat Commun 13:2561. doi:10.1038/s41467-022-30269-9 35538097 PMC9090908

[90] Néron B, Denise R, Coluzzi C, Touchon M, Rocha EPC, Abby SS. 2023. MacSyFinder v2: improved modelling and search engine to identify molecular systems in genomes. Peer Community Journal 3. doi:10.24072/pcjournal.250

[91] Wick RR. 2025. Verticall. Available from: https://github.com/rrwick/verticall

[92] Minh BQ, Schmidt HA, Chernomor O, Schrempf D, Woodhams MD, von Haeseler A, Lanfear R. 2020. IQ-TREE 2: new models and efficient methods for phylogenetic inference in the genomic era. Mol Biol Evol 37:1530–1534. doi:10.1093/molbev/msaa015 32011700 PMC7182206

[93] Hoang DT, Chernomor O, von Haeseler A, Minh BQ, Vinh LS. 2018. UFBoot2: Improving the ultrafast bootstrap approximation. Mol Biol Evol 35:518–522. doi:10.1093/molbev/msx281 29077904 PMC5850222

[94] Kalyaanamoorthy S, Minh BQ, Wong TKF, von Haeseler A, Jermiin LS. 2017. ModelFinder: fast model selection for accurate phylogenetic estimates. Nat Methods 14:587–589. doi:10.1038/nmeth.4285 28481363 PMC5453245

[95] Letunic I, Bork P. 2024. Interactive tree of life (iTOL) v6: recent updates to the phylogenetic tree display and annotation tool. Nucleic Acids Res 52:W78–W82. doi:10.1093/nar/gkae268 38613393 PMC11223838

