# Supplementary material for: Evolutionary dynamics and virulence factor variability in invasive Streptococcus pyogenes in Norway, 2017−2023
Source: mSphere. 2026 Mar 11;11(3):e00775-25. doi: 10.1128/msphere.00775-25 (PMC13037414; doi:10.1128/msphere.00775-25)
Supplement: Legends — for supplemental material. [file msphere.00775-25-s0003.docx]

Supplementary Figure 1: **Annual incidence rates iGAS per age-group per 100,000 population in Norway, 2017-2023.**

Supplementary Figure 2: **Phylogenetic tree of all 1163 isolates included in this study.** *Emm* types and *emm* cluster, which is a typing scheme used by the Center for Disease Control (CDC) (46) are annotated on the outside of the ring, showing the distinctness and clonality of *emm* types. Also shown is the presence of macrolide and tetracycline resistance genes. The number of contigs each genome was resolved to can be seen as black lines, showing that some *emm* types (such as *emm87*), contain more repetitive elements and will therefore assemble into a higher number of contigs.

Supplementary Table 1: **Metadata for all 1163 isolates included in this study.**

Supplementary Table 2: **Distribution of *speC*-*spd1*-carrying phages across *emm* types.**

Supplementary Table 3: **Distribution of phage defense systems across *emm* types.**
