# Supplementary material for: Evolutionary dynamics and virulence factor variability in invasive Streptococcus pyogenes in Norway, 2017−2023
Source: mSphere. 2026 Mar 11;11(3):e00775-25. doi: 10.1128/msphere.00775-25 (PMC13037414; doi:10.1128/msphere.00775-25)
Supplement: Table S2 — Phage presence by emm type. [file msphere.00775-25-s0005.docx]

**Table S2**  Distribution of *speC*-*spd1*-carrying phages across *emm* types.

| **Phage name** | **Accession** | ***emm1*** | ***emm4*** | ***emm12*** | ***emm28*** | ***emm87*** | ***emm89*** | **Total** |
| --- | --- | --- | --- | --- | --- | --- | --- | --- |
| Javan448 | MK448942.1 |  | 2.9% |  |  |  |  | 0.2% |
| Javan460 | MK448949.1 |  |  | 1.3% |  |  |  | 0.2% |
| Javan493 | MK448778.1 | 0.6% |  |  |  | 69.2% | 1.9% | 4.7% |
| Javan501 | MK448782.1 |  | 41.4% |  |  | 1.9% |  | 3.6% |
| Javan506 | MK448965.1 | 6.4% |  | 20.6% |  |  |  | 6.6% |
| Javan516 | MK448970.1 |  |  |  | 97.9% |  |  | 11.1% |
| Javan526 | MK448975.1 |  |  | 72.5% |  |  |  | 13.7% |
| Ola-1 | TBD |  | 51.4% |  |  |  |  | 4.3% |
| Ola-2 | TBD |  |  |  | 1.0% |  |  | 0.1% |
| Ola-3 | TBD |  |  |  |  |  | 81.5% | 10.4% |
| Ola-5 | TBD |  | 1.4% |  |  |  |  | 0.1% |
| Unresolvable | NA | 0.3% | 2.9% | 1.9% | 1.0% |  | 0.9% | 0.9% |
| Absent | NA | 92.8% | 0.0% | 3.8% | 0.0% | 28.8% | 15.7% | 42.2% |
