# Supplementary material for: Evolutionary dynamics and virulence factor variability in invasive Streptococcus pyogenes in Norway, 2017−2023
Source: mSphere. 2026 Mar 11;11(3):e00775-25. doi: 10.1128/msphere.00775-25 (PMC13037414; doi:10.1128/msphere.00775-25)
Supplement: Table S3 — Phage defense systems. [file msphere.00775-25-s0006.docx]

**Table S3**  Distribution of phage defense systems across *emm* types.

| **Type** | **System** | ***emm1*** | ***emm4*** | ***emm12*** | ***emm28*** | ***emm87*** | ***emm89*** |
| --- | --- | --- | --- | --- | --- | --- | --- |
| RM | RM type I | 100% | 100% | 100% | 100% | 100% | 100% |
| CRISPR-Cas | Class I subtype-I-C | 100% | 100% | 100% | 100% | 0% | 100% |
| CRISPR-Cas | Class II subtype-II-A | 100% | 100% | 100% | 100% | 0% | 100% |
| Abi | AbiD | 0% | 0% | 2.5% | 0% | 5.8% | 37% |
| Septu | Septu | 0% | 97.1% | 0% | 0% | 0% | 0% |
| SoFic | SoFic | 0% | 100% | 26.9% | 0% | 100% | 0% |
| VP1853 | VP1853 | 100% | 0% | 100% | 0% | 0% | 0% |
